# The In Vitro Interaction of 12-Oxophytodienoic Acid and Related Conjugated Carbonyl Compounds with Thiol Antioxidants

**DOI:** 10.3390/biom11030457

**Published:** 2021-03-18

**Authors:** Daniel Maynard, Andrea Viehhauser, Madita Knieper, Anna Dreyer, Ghamdan Manea, Wilena Telman, Falk Butter, Kamel Chibani, Renate Scheibe, Karl-Josef Dietz

**Affiliations:** 1 Department of Biochemistry and Physiology of Plants, Faculty of Biology, University of Bielefeld, 33615 Bielefeld, Germany; daniel.maynard@uni-bielefeld.de (D.M.); andrea.viehhauser@uni-bielefeld.de (A.V.); madita-knieper@web.de (M.K.); anna.dreyer@uni-bielefeld.de (A.D.); ghamdan.manea@uni-bielefeld.de (G.M.); wilena88@googlemail.com (W.T.); kamel.chibani@uni-bielefeld.de (K.C.); 2 Institute for Molecular Biology, Johannes Gutenberg-University Mainz, 55128 Mainz, Germany; F.Butter@imb-mainz.de; 3 Department of Plant Physiology, Faculty of Biology and Chemistry, Osnabrück University, 49069 Osnabrück, Germany; Renate.Scheibe@Biologie.Uni-Osnabrueck.DE

**Keywords:** *Arabidopsis thaliana*, cysteine covalent modification, phytohormones, protein–ligand interaction, peroxiredoxin, cyclophilin, thiol antioxidants, thioredoxin

## Abstract

α,β-unsaturated carbonyls interfere with numerous plant physiological processes. One mechanism of action is their reactivity toward thiols of metabolites like cysteine and glutathione (GSH). This work aimed at better understanding these interactions. Both 12-oxophytodienoic acid (12-OPDA) and abscisic acid (ABA) conjugated with cysteine. It was found that the reactivity of α,β-unsaturated carbonyls with GSH followed the sequence trans-2-hexenal < 12-OPDA ≈ 12-OPDA-ethylester < 2-cyclopentenone << methyl vinylketone (MVK). Interestingly, GSH, but not ascorbate (vitamin C), supplementation ameliorated the phytotoxic potential of MVK. In addition, 12-OPDA and 12-OPDA-related conjugated carbonyl compounds interacted with proteins, e.g., with members of the thioredoxin (TRX)-fold family. 12-OPDA modified two cysteinyl residues of chloroplast TRX-f1. The OPDAylated TRX-f1 lost its activity to activate the Calvin–Benson-cycle enzyme fructose-1,6-bisphosphatase (FBPase). Finally, we show that 12-OPDA interacts with cyclophilin 20-3 (Cyp20-3) non-covalently and affects its peptidyl-prolyl-cis/trans isomerase activity. The results demonstrate the high potential of 12-OPDA as a diverse interactor and cellular regulator and suggest that OPDAylation may occur in plant cells and should be investigated as novel regulatory mechanism.

## 1. Introduction

12-oxophytodienoic acid (C_18_H_28_O_3_, 12-OPDA) is synthesized from α-linolenic acid (C_18_H_30_O_2_) by a short enzyme cascade in the chloroplast and functions both as a signaling molecule by itself and as a precursor for the plant hormone jasmonic acid (JA). 12-OPDA has a 2-cyclopentenone moiety (CP) which reacts with the thiol-peptide glutathione (GSH) in a 1,4-Michael addition reaction [1]. Michael reactions occur between a nucleophilic agent (Michael donor), such as thiol molecules, and an electrophilic compound (Michael acceptor). An example of Michael acceptors are conjugated carbonyl compounds (CCCs). They function as reactive electrophilic species (RES) and participate in biochemical scavenging reactions with thiol groups. It has been described that the main low-molecular weight thiol in plants, namely GSH, forms Michael adducts with a wide spectrum of biologically active CCCs such as 2-trans-hexenal (C_6_H_10_O, HEX), an aldehyde that, as its C-18 ketone counterpart, is derived from the octadecanoid pathway, or methyl vinylketone (C_4_H_6_O, MVK) [2,3].

The highly toxic MVK forms by non-enzymatic oxidation of isoprene (C_5_H_8_) or poly-unsaturated fatty acids (PUFA) [4,5]. Isoprene is synthesized from the 2-C-methylerythritol-5-phosphate pathway that delivers precursors for many plant metabolites including the plant hormone abscisic acid (ABA, C_15_H_20_O_4_) [6]. Previous studies revealed that ABA synthesis is enhanced by the only thiol-bearing amino acid cysteine (Cys, C_3_H_7_NO_2_S), whereas 12-OPDA stimulates the synthesis of O-acetyl serine (OAS) and subsequently Cys and GSH [7,8]. Both, 12-OPDA and ABA, are enzymatically derived α,β-unsaturated cyclic ketones and it appeared interesting to investigate their interaction with Cys (for information, see Table 1 and Figure S1).

12-OPDA is exported from the chloroplast to the cytosol via the ABA receptor-related protein JASSY with affinities highly dependent on the microenvironment being 1 mM in hydrophilic solutions and 11 µM in hydrophobic environment such as in membranes [13]. The half-life of 12-OPDA is estimated to be ~26 min at typical GSH concentrations of 1 mM [14]. Concomitantly, 12-OPDA concentrations reach micromolar levels, thus potentially exceeding typical hormone concentrations. Concentrations reaching mM levels have been reported for other CCCs [15,16]. The mammalian counterparts of 12-OPDA, the prostaglandins 15dPGJ2 and PGJ2, reach concentrations of 80 µM [12]. Prostaglandins exert direct and indirect signaling effects as hormone and reactive electrophilic species (RES).

These features appear to be similar to those of 12-OPDA. The 12-OPDA–cyclophilin 20-3 (Cyp20-3) interaction in plants [7] can be considered as a ligand-receptor interaction and results in enhanced thiol synthesis, similar to the 15dPGJ_2_-Keap-Nrf2 module in humans [11]. In humans, accumulating CCCs undergo covalent interactions with thiol redox-buffers such as GSH; consequently, the redox potential and oxidative stress increases allowing reactive oxygen species (ROS)-sensing proteins such as 2-cysteine peroxiredoxin (2-CysPRX) to exert their function in signaling. In this scenario, RES-detoxifying enzymes such as 2-alkenal reductases also play a key role in redox balancing [10].

However, the reaction network of CCCs in the biological context is far from fully explored. To improve our understanding, this work addresses hypothetical “novel” interactions of 12-OPDA and related compounds with proteins as suggested [9,10,14] similar to the recent examples of the interaction of 15-dPGJ_2_ with the ROS and RES-buffering serum albumin [17] and the redox signal mediating enzyme thioredoxin (TRX) [18]. The main purpose of this study was to provide in vitro data on the interaction of 12-OPDA with selected redox-associated plant thiol antioxidants and to compare these with other CCCs and 12-OPDA-related compounds.

## 2. Materials and Methods

### 2.1. Chemicals

CP (C112909), CPa (C112402), GSH (G4251), Cys (C7352), N-succinyl-Ala-Ala-Pro-Phe p-nitroanilide (S7388), α-chymotrypsin (C4129), phosphoglucose isomerase (P5381), DTNB (D8130) were purchased from Sigma-Aldrich (Darmstadt, Germany), MVK (128000050), HEX (158130250) from Acros-Chemicals (Thermo Fisher Scientific, Geel, Belgium), abscisic acid (A0941) from Duchefa Biochemie (Haarlem, Netherlands), glucose-6-phosphate dehydrogenase (10737232001) from Roche (Mannheim, Germany). Serine (1714. 2), ASC (3525. 2) and NADPH (AE14. 3) were purchased from Roth (Karlsruhe, Germany) and H_2_O_2_ (23615.261) from VWR (Langenfeld, Germany). NADP (A1394.0001) and DTT (A1101.0100) were purchased from Applichem (Darmstadt, Germany). 12-OPDA was synthesized as described [19], 12-OPDA-Et was a generous gift of Prof. Harald Gröger from the Organic Chemistry and Biotechnology Department at University Bielefeld. All other chemicals and organic solvents were purchased from diverse commercial suppliers and were of analytical grade.

### 2.2. Data Processing, Databases and in Silico Studies

Data processing, calculations and presentations were performed using Microsoft Office 2016. Statistical analysis was performed using the creative commons online calculation tool (https://astatsa.com/OneWay_Anova_with_TukeyHSD/, 2016 Version, Creative Commons, Navendu Vasavada accessed on 28 February 2021). 3-D model structures were generated using the SwissProt online tool (https://swissmodel.expasy.org/interactive, accessed on 28 February 2021) [20]. TRX-f1 (Uniprot/SwissProtID: Q9XFH8) and TRX-m1 (Uniprot/SwissProtID: O48737) were fitted to the structure PDB: 2PU9 of TRX-f1 from *Spinacia oleracea* as the template (75.9% identity with Txf1, 32.0% identity with TRX-m1). The accuracy of the protein structure models was predicted by the SWISS MODEL server with Global Model Quality Estimation (GMQE) scores of 0.77 (TRX-f1), 0.68 (TRX-m1) and QMEAN values of -0.48 (TRX-f1) and -1.92 (TRX-m1). As for 2-CysPRX, the 2-CysPRX-C54S structure was used as a template (PDB: 5ZTE, 99.48% identity). Generated models were freed from water molecules and heteroatoms prior to in silico studies. Structural overlay of TRX-m1, TRX-f1 and 2-CysPRX models was performed using the default align command of Pymol [21]. Blind docking of TRX-f1 model with 12-OPDA (ZINC4095588) via the SwissDock online server (http://www.swissdock.ch/docking, accessed on 28 January 2021) was performed with default settings [22]. Blind-docking studies of Cyp20-3 Uniprot/SwissProtID: P34791 with 12-OPDA, MVK, CP, CPa and 7-iso JA (JA) were performed with a Cyp20-3 model based on human Cyp (Uniprot/SwissProtID: P62937), PDB: 5KUO, 66.87 identity (GMQE score of 0.43 and a QMEAN value of −0.26) and respective ZINC entries (https://zinc.docking.org/, accessed on 28 January 2021) [23] ZINC4095588, ZINC1680420, ZINC95918567, ZINC895304, ZINC4492884 as described for TRX-f1 docking study. All clusters were analyzed and depicted with the molecular visualization software UCSF-Chimera [24]. Additionally, blind-docking of TRX-f1 and Cyp 20-3 models with organic compounds was carried out using the freely available CB-Dock server (http://clab.labshare.cn/cb-dock/php/, accessed on 26 February 2021) using default settings and structure data file (SDF) formats of ligands [25]. Information on chemicals was received by the open online databases Pubchem [26] (pubchem.ncbi.nlm.nih.gov, accessed on 4 December 2020) and Molinspiration [27] (molinspiration.com, accessed on 4 December 2020) as indicated in the text. Molar extinction coefficients at 280 nm of proteins were calculated using the Expasy Protparam tool (https://web.expasy.org/cgi-bin/protparam/protparam, accessed on 28 February 2021) [28].

### 2.3. Synthesis and Detection of Cys-12-OPDA

For synthesis and detection of the 12-OPDA adduct, 10 µL of 12-OPDA (34 mM in MeOH) were added to 0.34 µmol Cys dissolved in 20 µL 40 mM Na-P_i_, pH 6.2 and incubated for 120 min at 30 °C. Finally, 70 µL MilliPore water (MPW) was added to the reaction mixture and directly injected into the ESI-MS aperture. The ESI-MS spectrum was recorded via Bruker Daltonics Esquire 3000 (Bruker Daltonik, Bremen, Germany) with the standard ESI ion source in positive mode. Mass spectroscopy conditions were set as follows: drying gas, nitrogen (4 L min^−1^, 300 °C); nebulizer gas, nitrogen (5 psi); capillary voltage, 2–6 kV; vaporizer temperature, 300 °C. Spectra were collected using an electrospray ionization source operating in the positive mode and data were processed with Esquire control data qualitative analysis software. As controls Cys was added to 10 µL MeOH and 20 µL 40 mM Na-P_i_, pH 6.2, and 12-OPDA was added to 20 µL 40 mM Na-P_i_, pH 6.2. The reactions were incubated for 120 min at 30 °C and mixed with 70 µL MPW. Analysis was carried out as described above. The standard calibration kit (ESI-T Tuning Mix, Agilent Technologies, Part. Nr G2431A, Agilent Technologies, Santa Clara, CA, USA) was used as described in the manufacturing manual in order to correctly assign the mass charge ratios of ions presented in this article.

### 2.4. Mass Spectrometric and Calorimetric Detection of Cysteine Interacting with Abscisic Acid

Conditions and chemicals for identifying ABA-Cys adduct via MS were adjusted as in Section 2.3, except for the following changes. ABA solution (20 µL, 5 mM in EtOH) was mixed with 1 mg Cys and allowed to react for 5 min at 30°C, followed by addition of 20 µL 40 mM Na-P_i_, pH 7.2, and incubation for 5 min at 30°C. To the sample, 60 µL MPW was added, diluted 1:1 in 50% isopropanol and injected into ESI-MS at 180 µL min^−1^, with target mass set at 300. Control analyses of ABA and Cys were performed. For MS settings, see Section 2.3. Isothermal titration microcalorimetry (ITC) was performed using the MicroCal LLC ITC (MicroCal LLC, Northampton, MA, USA) instrument. A solution of 5 mM ABA was injected into an adiabatically thermostated cuvette containing 0.5 mM Cys or Ser. Amino acids and ABA were dissolved in 40 mM K-P_i_, pH 8.0. The control titration was performed by injecting 5 mM ABA into buffer (40 mM K-P_i_, pH 8.0) or buffer into 0.5 mM Cys. The parameters for each injection were set to 5 µL injection volume, 10 s duration, spacing between injections to 120 s, 304 rpm stirring speed, cell temperature to 25 °C, reference power 30 and high feedback.

### 2.5. Analysis of Free Thiol Content of GSH Incubated with CCCs

GSH (180 µL 1 mM in 40 mM K-P_i_, pH 6.6, 7.2 or 8.0) was mixed with the same volume of 12-OPDA and other chemicals (see results) at 1 mM concentration (in 40 mM K-P_i_, pH 6.6, 7.2 and 8.0) at 25 °C on a shaker at 1500 rpm for 30 min (Ika, VXR basic vibrax, Fisher Scientific, Schwerte, Germany). The control sample was obtained as above with buffer instead of chemicals, containing 1% EtOH. Samples were analyzed for thiol content by mixing 40 µL of the incubation mixture, GSH stocks (0 mM, 0.125 mM, 0.25 mM, 0.5 mM in 40 mM K-P_i_ at respective pH) with 160 µL master mix (140 µL 120 mM Na-P_i_, pH 7.8, 5 mM EDTA, and 20 µL 6 mM DTNB in 120 mM Na-P_i_, pH 7.8) at room temperature. The absorbance was measured at 412 nm after 5 min of incubation in the dark using the Biotek KC4 microplate reader.

### 2.6. Effect of Exogenously Applied Methylvinyl Ketone (MVK) Incubated with Thiol and Non-thiol Antioxidant on Photochemical Activity of Leaf Discs

MVK (0.5 mL 5 mM, in 40 mM K-P_i_, pH 8.0) or 40 mM K-P_i_, pH 8.0, (control) was added to 0.5 mL of 5 mM ASC or GSH in 40 mM K-P_i_, pH 8.0 and mixed (100 rpm) for 11 h at 25 °C. Leaf discs of 5 mm diameter were punched out from five rosettes of six-week old climate chamber-grown *A. thaliana (Col-0)*. The discs were immediately placed upside-down on 4 mL 100 µM CaCl_2_ solution and measured for initial effective quantum yield of PS II (ΔF Fm´^−1^: ΦPSII) value (*t* = 0) using the pulse amplitude modulated chlorophyll fluorimeter (MiniPAM, Walz, Effeltrich, Germany). A total of 1 mL of treatment or control solution was added and mixed carefully without flipping the leaf discs. The samples were exposed to light of 100 μmol photons m^−2^s^−1^ at 25 °C. ΦPSII was taken as (Fm´–F) Fm´^−1^, where F is the fluorescence yield of the light-adapted sample in the steady state and Fm´ is the maximum fluorescence yield after applying a saturating light pulse of 800 ms duration at an intensity of 3000 µmol photons m^−2^s^−1^ [29].

### 2.7. Synthesis and Purification of FBPase, TRX-f1, TRX-m1, 2-CysPRX, NTRC, Cyp20-3 and Cyp20-3 Variants

His_6_-tagged proteins of FBPase, 2-CysPRX, TRX-m1 and TRX-f1 and NTRC without transit peptides were generated and produced as described in [30,31]. The pET28a expression vector encoding Cyp20-3 and Cyp20-3 cysteine-serine variants was transformed into Nico21(DE3) cells (NEB) for heterologous expression and protein generation in *Escherichia coli* (*E. coli* )as described [19] with omission of His_6_-peptide cleavage step. Cys-Ser variants of Cyp20-3 were generated with Cyp20-3 as template and in vitro mutagenesis primers [32]. The preparation of proteins for PPiase activity required aliquots (Cyp20-3 WT and Cys-Ser variants) of high protein concentration (4 mg mL^−1^ stocks) and no pre-treatment with reductants. Therefore, the following minor modifications were introduced. Protein expression was induced with 0.9 mM isopropyl β-D-1-thiogalactopyranoside (IPTG). Organic additives except for imidazole were omitted in lysis, washing and elution buffers. Proteins were concentrated using a Vivaspin™ 20 column (Sartorius, Göttingen, Germany) at 3500× *g* and 4 °C for 30–90 min. Samples were dialyzed in 50 mM Na-P_i_, pH 8.0. Protein concentrations were determined spectrophotometrically using molar extinction coefficient at 280 nm of 17085 M^−1^ cm^−1^ for TRX-f1, 21095 M^−1^ cm^−1^ for TRX-m1, 20065 M^−1^ cm^−1^ for 2-CysPRX, 38195 M^−1^ cm^−1^ for FBPase and 12950 M^−1^ cm^−1^ for Cyp20-3. As for ITC (Section 2.13) and activity assays (Section 2.14) with Cyp20-3 and its variants and NTRC (see Section 2.12), proteins were quantified using BSA as standard as described [33]. Protein purity (>90%) was assessed via 14% SDS-PAGE by loading equal protein amounts.

### 2.8. Fluorescence Properties of TRX-Fold Proteins and Cyp20-3

Measurements were carried out at concentrations of 17.5 µM for the reduced (TRX-f1red) and 18.3 µM for the untreated TRX-f1 protein (TRX-f1). Analysis of TRX-m1 and 2-CysPRX in untreated or reduced form at concentrations of 18.3 µM was also performed. Reduced proteins were obtained as described (see Section 2.11). The ligand stock solution contained 34 mM 12-OPDA in methanol. Ligands were diluted with methanol. Then, 150 µL of TRX-f1 was incubated with 1.5 µL of 12-OPDA (34 mM) or 1.5 µL methanol serving as control (TRX-f1 red and TRX-f1) at 25 °C for 10 min. The reduced protein was incubated with 1.5 µL of 12-OPDA (17 mM) to obtain ligand/protein ratios comparable to the TRXf1 measurement. Then, 120 µL of the clear solutions were transferred into a 1 cm quartz cuvette and emission spectra were recorded using a spectrofluorometer (SFM 25; Kontron Instruments) at 25 °C. The excitation wavelength was set to 280 nm, the scan rate to 1 nm sec^−1^ and the high voltage (HV) to 450 V. Due to high intrinsic fluorescence of 2-CysPRX, HV was set to 360 V. The emission spectrum was recorded as relative fluorescence in the spectral range of 300–400 nm. Comparative fluorescence analysis of TRX-fold proteins at high protein concentration was performed using 36 µM solutions of reduced protein incubated with indicated 12-OPDA concentrations for 60 s. Settings were the same as above except the emission spectra were recorded in the spectral range of 310–355 nm. Fluorometric analysis of Cyp20-3 incubated with 12-OPDA, MVK, CPa and CP (see Table 2) was performed as follows. Then, 15 mM of test compound dissolved in EtOH was mixed with 40 mM K-P_i_, pH 8.0, to a final concentration of 1.5 mM. The control solution was prepared identically, but without added compound. Then, 10 µL of freshly prepared 1.5 mM compound or control solution (10% EtOH in 40 mM K-P_i_, pH 8.0) were incubated for 60 s with 110 µL of 0.67 mg mL^−1^ Cyp20-3 at 25 °C in a quartz cuvette and directly recorded for fluorescence emission spectra (SFM 25; Kontron Instruments) at 25 °C. The excitation wavelength was set to 280 nm, the scan rate to 1 nm sec^−1^, HV to 450 V and the range to 310–400 nm. All ligands did not cause any interference with background.

### 2.9. Covalent Modification of TRX-f1 by 12-OPDA for Analysis by Mass Spectrometry

Samples were processed for mass spectroscopy with Thermo Scientific Q Exactive Plus Hybrid Quadrupole Orbitrap mass spectrometer as follows. For reduction, TRX-f1 (TRX-f1 red) was incubated with 500 µM DTT for 30 min at room temperature followed by a desalting step using PD 10 column and 30 mM Tris-HCl, pH 7.9, as elution buffer. Similar to the protocol of Shibata [18] using a ligand/protein ratio of ~12, TRX-f1red was incubated with 10-fold molar excess of 12-OPDA at 35 °C for 10 min. As a control, TRX-f1red was incubated with the same volume of methanol. The end concentration of methanol in protein samples was below 1% if not stated otherwise. Then, 25 μL of the samples (15 μg) were desalted, eluted in 30 μL of 80% acetonitrile and 0.1% formic acid and diluted with 470 μL 50% methanol and 0.1% formic acid. Q Exactive Plus intact protein mode was activated and sample injected at 20 μL min^−1^ (ESI Source). Myoglobin (5 pmol µL^−1^) was measured as a standard and the resolution was 140,000. Results derive from measuring the same samples at different modes, these were in-source 0, 20, 30 and 40. Deconvoluted masses were calculated as neutral masses.

### 2.10. FBPase Activity Test

FBPase activity was measured using a Shimadzu UV-Vis spectrophotometer PC-UV 2401 and a quartz cuvette (1 cm) based on the assay described in [30]. The assay was performed in buffer (30 mM Tris-HCl, 5 mM MgSO_4_, pH 8.0) containing 0.2 mM NADP, 1 U glucose-6-phosphate dehydrogenase, 1 U phosphoglucose isomerase and 20 µg of recombinant FBPase (0.5 µM). The total reaction volume was 1 mL. After 40 s of baseline recording, the reaction was started by adding fructose-1,6-bisphosphate at a final concentration of 0.6 mM. For the experiment, 5 µM of recombinant TRX-f1 with or without 12-OPDA (preparation as described in Section 2.9) was mixed with the substrate prior to addition to the quartz cuvette. Absorption was recorded at 340 nm and 25 °C. Control experiments were performed with or without TRX-f1 (5 µM) alone. If necessary, the same amount of the appropriate solvent was used.

### 2.11. Protein Thiol Estimation with DTNB

Proteins were reduced (1 mM DTT, 30 min, 37 °C) and desalted using a PD10 column (Sigma Aldrich, Taufkirchen, Germany) to remove excess of DTT. Free thiols were determined with 5,5′-dithiobis-(2-nitrobenzoic acid) (DTNB) as described in [31,34]. Then, 150 µL of reduced proteins TRX-f1 (22 µM), TRX-m1 (22 µM), 2-CysPRX (22 µM) or Cyp20-3 (20 µM) were mixed with 1.5 µL of either 30 mM, 15 mM or 7.5 mM stock solution of 12-OPDA or MVK (in EtOH) or EtOH as control. After 15 min of incubation at 37 °C, samples were mixed with 10 µL DTNB (2 mM in 40 mM K-P_i_, pH 8.0) and incubated for 30 min in darkness to estimate the free thiols. Samples were measured at 412 nm, ε_412nm_ = 13,600 M^−1^ cm^−1^ (Shimadzu 2401, quartz cuvette, 25 °C) for determination of thiol/protein ratio with background correction (150 µL 40 mM K-P_i_, pH 8.0, 10 µL 2 mM DTNB, 1.5 µL ligand stocks or EtOH).

### 2.12. NTRC-Dependent Peroxidase Activity of 2-CysPRX in Presence of CCCs

NTRC-dependent 2-CysPRX activity was measured using a Shimadzu UV–Vis spectrophotometer PC-UV 2401 at 25 °C as follows. In a quartz cuvette (1 cm), 90 µL 50 mM K-P_i_, pH 7.5, was mixed with 1 µL CPa, or the indicated CCC stock solutions (all dissolved in EtOH), followed by addition of 10 µL 2-CysPRX (1 µg µL^−1^) and 15 µL NTRC (2 µg µL^−1^). After 20 s, 5 µL NADPH (3 mM stock in buffer) was mixed in the cuvette. The baseline was measured at 340 nm for 3 min before the reaction was initiated by adding 10 µL 980 µM H_2_O_2_ or the same volume of H_2_O as a control, similar as described in [35]. 2-CysPRX peroxidase activity was coupled to NTRC-dependent NADPH oxidation (initial slope taken 20 s after H_2_O_2_ addition) using the extinction coefficient of 6220 M^−1^ cm^−1^. No change of baseline occurred when BSA instead of 2-CysPRX was used, or when NADPH or NTRC were omitted from the reaction.

### 2.13. ITC-Analysis of Interaction between 12-OPDA and Cyp20-3

Recombinant Cyp20-3 WT and C171S-variant were dialyzed overnight in 40 mM K-P_i_, pH 8.0, twice against 1000-fold excess volume at 4 °C. Proteins (28 µM) were degassed in a Thermovac (Northhampton, MA, USA) at 20 °C for 3 min and filled in the ITC cell (MicroCal LLC, Northampton, MA, USA). 12-OPDA (500 mM in EtOH) was diluted into degassed dialysis buffer (3 min at 20 °C) to minimize background interference during injection of 12-OPDA (500 µM) in the filled cuvette. The injection volume was 7 µL, duration 14 sec, time interval between injections 90 sec, stirring speed 264 rpm, T 25 °C, reference power 15, feedback high, fast equilibration auto. The control titration was performed by injecting 0.1% EtOH in dialysis buffer (degassed as described) in the same protein solutions. For quantifying binding characteristics, control titrations were subtracted from ligand protein ITC data and integrated heat responses were fitted to the single binding site model using the ORIGIN software package supplied with the calorimeter.

### 2.14. Activity Assays for Cyp20-3 in the Presence of 12-OPDA

Cyp20-3 protein was either oxidized by overnight dialysis at 4 °C twice in 1000-fold volume of 50 mM Na-P_i_, pH 8.0, containing 10 mM H_2_O_2_ or reduced using an immobilized reductant column (Thermo-Scientific). The catalytic peptidyl-prolyl-*cis*/*trans*-isomerase (PPIase) efficiency expressed as K_cat_/Km was measured as in [32] using a Cary 3500 UV–Vis spectrophotometer (Cary 3500 UV-Vis, Agilent, Santa Clara, CA, USA) and a 2.5 mL polystyrene cuvette. 100 mM of substrate (N-succinyl-Ala-Ala-Pro-Phe p-nitroanilide) was mixed with varying amounts of pre-oxidized or reduced Cyp20-3 (5, 10, 25, 50, 75, 100 nM) in 35 mM HEPES, pH 8.0. After adding 26 µM of 12-OPDA, the sample was incubated at 8 °C and 800 rpm for 10 min. The control solution lacked Cyp20-3. The baseline was recorded at 390 nm for 3 min before the reaction was initiated by adding 0.8 mg α-chymotrypsin. Catalytic efficiencies of Cyp20-3 C54S and C171S could not be measured because of protein precipitation at the required concentration of 4 µg µL^−1^ in the activity test.

## 3. Results

### 3.1. Reactivity of Michael Systems with Cysteine, Glutathione and Ascorbate

**Figure 1:**
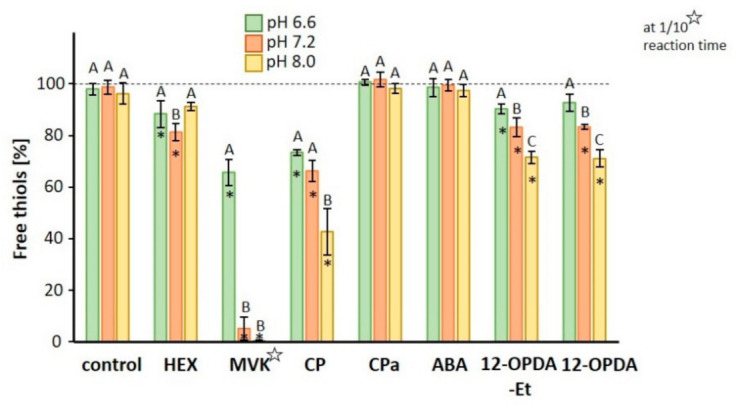
Reactivity of 12-OPDA and related molecules with glutathione (GSH) at three physiological pH values. 100% refers to 500 µM GSH in the sample, 50% GSH corresponds to 250 µM GSH being conjugated to the indicated compound in a reaction period of 30 min at 25 °C. The reaction time with methyl vinylketone (MVK) was 3 min (indicated with a star). Results are presented as means ± SD (n = 6–9). Asterisks indicate a statistically significant difference between buffer control and treated samples at respective pH-values. Significant differences of treatments or buffer control between different pH-values are indicated by different letters A–C. Significance of difference (*p* ≤ 0.05) was calculated using one-way ANOVA with post hoc Tukey HSD test. For details, see Section 2.

The first set of experiments explored the reactivity of 12-OPDA and related molecules (500 µM) with GSH (500 µM) at three physiological pH values (Figure 1). Of the seven tested compounds (molecular structures see Figure S1), MVK was most reactive followed by 2-cyclopentenone (CP), 12-OPDA and HEX. It is interesting to note that the ethyl-esterified 12-OPDA-Et displayed the same reactivity as 12-OPDA and that in all examined reactions except for HEX, the reactivity positively correlated with pH-value as expected with increasing deprotonation of the thiol. The thiol depletion of GSH in the presence of 12-OPDA and 12-OPDA-Et was 7% vs. 10% at pH 6.6, 17% for both at pH 7.2 and 28% vs. 29% at pH 8.0.

**Figure 2:**
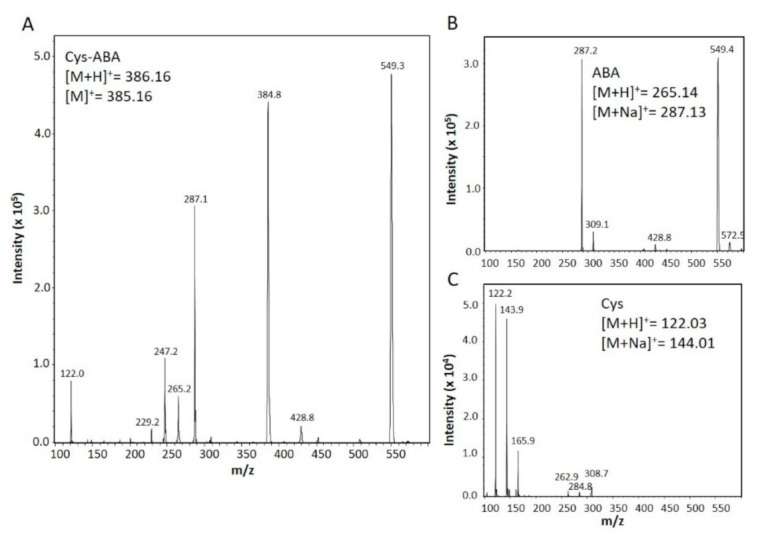
Mass spectrometric analysis of the interaction product of abscisic acid (ABA) with Cys. MS spectra of the ABA–Cys mixture (**A**) revealed adduct formation at 384.8 *m*/*z*. This peak was not observed with ABA (**B**) or Cys (**C**). ESI-MS spectra were recorded with the Bruker Daltonics Esquire 3000 in positive ionization mode. For details, see Section 2.

As expected, the saturated cyclopentanone (CPa), mimicking the cyclic head unit of jasmonates, did not react with GSH, whereas ABA led to insignificant thiol modification. Due to its CCC characteristics, we expected thiol modification. After incubation of Cys with ABA, a new compound appeared (*m*/*z* positive ionization mode C_18_H_27_O_6_NS+ expected: 385.16, observed: 384.80) (Figure 2). Despite the deviation by 0.36 mass units, we propose the reaction to proceed at equimolar ratio. ITC analysis of ABA and Cys (Figure S2) revealed an exothermic reaction, no enthalpic response was recorded when ABA was injected in serine or buffer or when buffer was injected in Cys, confirming the interaction of ABA (C_15_H_20_O_4_) with Cys (C_3_H_7_NO_2_S).

**Figure 3:**
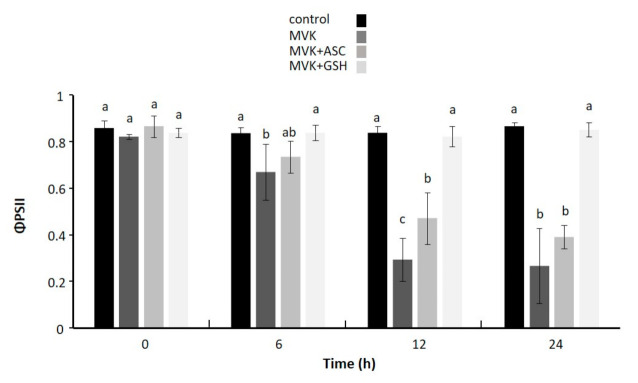
Photosynthetic performance of leaf discs floating on MVK (gray) pre-incubated with GSH or ASC. Changes in quantum yield of photosystem II (ΦPSII) of *A. thaliana* (Col-0) in response to treatment with methyl vinylketone (MVK), MVK preincubated with GSH (MVK+GSH) or ASC (MVK+ASC). Leaf discs floated on a solution containing the indicated compounds (500 µM) in 100 µM CaCl_2_ for membrane stabilization. ΦPSII was determined by chlorophyll a fluorescence analysis using the pulse-amplitude-modulated photosynthesis yield analyzer (Mini-PAM). Data are means ± SD, n = 5. Significant differences at *p* ≤ 0.05 were calculated using one-way ANOVA with post hoc Tukey HSD and are represented by letters a–c.

As demonstrated in this and previous studies, CCCs such as cyclopentenones or MVK react with GSH [2,14] and ascorbate (ASC) [9,36]. This chemical reaction may contribute to the protection of other cell constituents from damaging reactions with CCCs. To demonstrate the beneficial effect of ASC or GSH to ameliorate the toxic effects of CCCs, we monitored the photosynthetic performance of leaf discs floating upside-down on MVK solution supplemented with low molecular weight antioxidants at equimolar concentrations (500 µM) for 6 h, 12 h or 24 h under normal light conditions. The floating media was 40 mM K-P_i_, pH 8.0, to mimic physiological conditions and to ensure high Michael reactivity as inferred from our results (see Figure 1) and from literature on ASC chemistry [36]. As seen in Figure 3, MVK strongly inhibited photosynthesis already after 6 h, while leaf samples exposed to MVK+GSH maintained a high photosynthetic yield during the entire experiment. The detoxifying ability of ASC on MVK was insignificant after >6 h, revealing that under the tested conditions GSH ameliorated the toxic effects of excessive carbonyl magnitudes better than ASC. GSH and ASC had no effect on ΦPSII yield in comparison to untreated control.

### 3.2. The Interaction of 12-OPDA with Plant Thioredoxin

**Figure 4:**
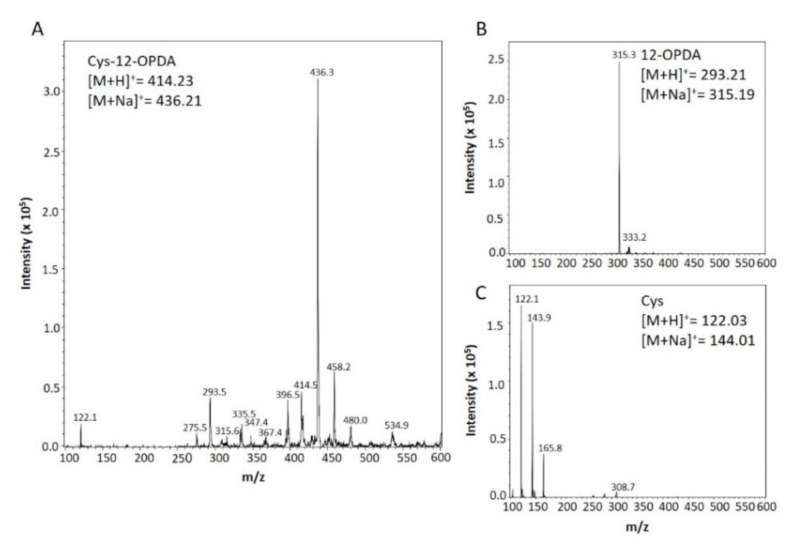
Identification of Cys-12-OPDA via mass spectrometry. (**A**) MS analysis of the product formed after incubation of Cys with 12-OPDA revealed Cys-12-OPDA as the main product. The incubation without addition of either 12-OPDA or Cys is also presented (**B**,**C**). The insets depict molecular masses and the expected *m*/*z* ratios of the indicated main products. ESI-MS spectrum was recorded via Bruker Daltonics Esquire 3000 in positive ionization mode. For details, see Section 2.

Proteins are known to interact with CCC [37,38]. In mammals, TRX is reported to be modified at cysteinyl residues by the prostaglandin 15dPGJ_2_ [18]. Due to the resemblances of 12-OPDA to 15dPGJ_2_ (see Table 1 and Figure S1) and human TRX to chloroplastic TRX-f1 [39], we proposed a similar chemical event as described in Shibata et al. [18] for the plant model Arabidopsis. The first experiments addressed the question of whether Cys, the common feature of targets, forms an adduct with 12-OPDA detectable by MS. The MS analysis of the 12-OPDA Cys incubation mixture (Figure 4) revealed a base peak with a mass charge ratio (*m*/*z*) of 436.3 indicating expected Michael adduct formation of the sodium stabilized 12-OPDA-Cys adduct (expected *m*/*z*=436.2 positive ionization mode). 

The mature TRX-f1 sequence without the annotated chloroplast targeting address carries three cysteines, of which two are in proximity to the tryptophan of the active site (WCGPCK motif) which are also present in both TRXs from human and plants. Motivated by the above result (Figure 4) and published fluorescence assays with TRX [40], we performed protein fluorescence studies with reduced (red) and untreated TRX-f1 in the presence of 12-OPDA. Protein fluorescence is mainly due to Trp residues and thus fluorometry seemed an appropriate method to investigate whether 12-OPDA addresses active site residues. 12-OPDA quenched the intrinsic protein fluorescence of both TRX-f1red and untreated TRX-f1 (Figure S3), suggesting that 12-OPDA binds in proximity to the WCGPCK motif that is structurally hardly affected in known crystal structures of redox altered TRXs ([41] and references cited therein).

**Figure 5:**
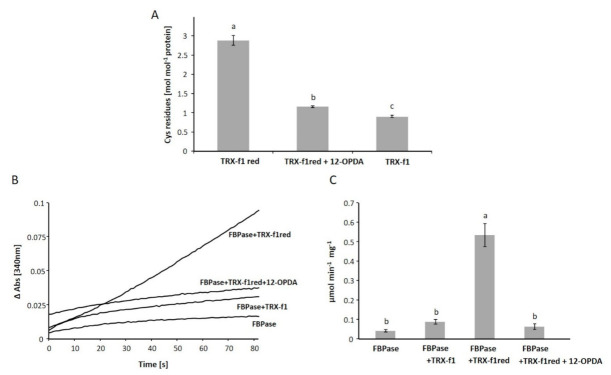
12-OPDA targets TRX-f1 and affects the activation of fructose 1,6-bisphosphatase (FBPase). (**A**) TRX-f1 thiols were modified by 12-OPDA. The number of thiols in proteins (22 µM) was detected via the DTNB assay for reduced or untreated TRX-f1 or after incubation of reduced TRX-f1 with 12-OPDA (300 µM). Data are means of n ≥ 3 ± SD. Significant differences at *p* ≤ 0.05 were calculated using one-way ANOVA with post hoc Tukey HSD and are represented by letters a–c. (**B**) The activation of FBPase by reduced TRX-f1 was inhibited after incubation with 12-OPDA shown by the FBPase activity test performed with reduced TRX-f1red without 12-OPDA and TRX-f1red with 12-OPDA incubation and untreated TRX-f1 (TRX-f1). The assay was performed 3 times with highly similar results, as seen in the respective calculated mean ± SD of FBPase activities shown in (**C**). Significant differences at *p* ≤ 0.05 were calculated using one-way ANOVA with post hoc Tukey HSD and are represented by letters a–b.

To study whether 12-OPDA covalently modifies Cys residues of TRX-f1, we quantified the accessible thiols [34] revealing that 12-OPDA binds free thiol residues in TRX-f1. The thiol content of TRX-f1red was about three moles per mol protein. Only about one mol thiol per mol protein was detected in untreated TRX-f1 (TRX-f1), and essentially also one mol thiol per mol protein in TRX-f1red after treatment with 12-OPDA (TRX-f1red+12-OPDA) (Figure 5A).

This analysis showed that untreated TRX-f1 contains a disulfide bridge between the catalytic and resolving Cys residues and adopts the oxidized form. To investigate the effect of 12-OPDAylation on the enzymatic activity of TRX-f1, we incubated reduced TRX-f1 with 12-OPDA at a molar ratio of 1:10, dialyzed the protein and then determined the FBPase activation as described in Vaseghi et al. [30] using recombinant FBPase. As seen in Figure 5B, TRX-f1red activated FBPase (0.53 ± 0.06 µmol min^−1^ mg^−1^ protein), while the activity of FBPase with OPDAylated TRX-f1 (0.062 ± 0.014 µmol min^−1^ mg^−1^ protein) was as low as FBPase with addition of untreated TRX-f1 (0.087 ± 0.012 µmol min^−1^ mg^−1^ protein) or without addition of its activator TRX-f1red (0.041 ± 0.006 µmol min^−1^ mg^−1^ protein).

**Figure 6:**
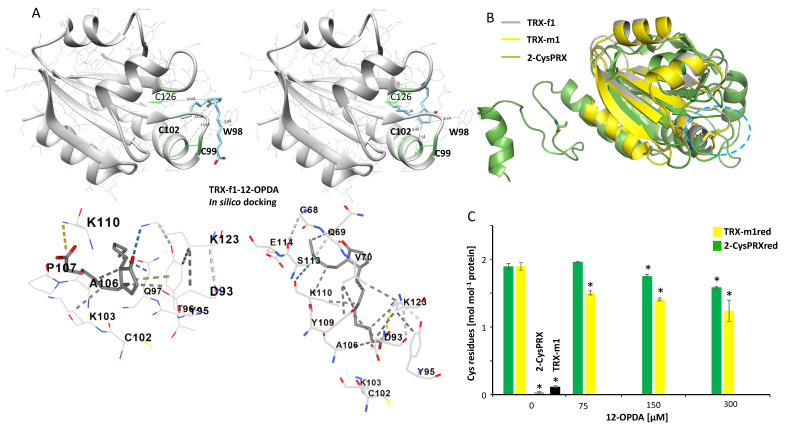
Modeling major TRX-fold proteins as 12-OPDA targets. (**A**) Models of the two best scored in silico docking results of TRX-f1 probed with 12-OPDA. The tertiary structure of TRX-f1 (grey) obtained by homology modeling based on TRX-f1 model PDB: 2PU9. The protein consists of five beta-strands and three alpha-helices. The 12-OPDA-binding region is suggested to be located in proximity to the active site Cys and Trp98. The left view suggests that Trp98 of TRX-f1 positions within 5 Å distance of the octanoic acid side chain of 12-OPDA, while the right view suggests that Cys 102 is 5.4 Å close to the electrophilic center of 12-OPDA. 12-OPDA is shown as blue stick model. Distances of sulfur atoms towards ß-C of 12-OPDA are indicated as dashed lines. Docking and visualization were performed using the online server SwissDock and the free accessible software package UCSF Chimera. The two graphics below show the first two top scoring docking results of TRX-f1 with 12-OPDA performed with CB-Dock. Suggested 12-OPDA (shown in gray stick presentation) binding cavities (sizes of 84 (left) and 153 (right), Vina scores of -5.5 (left) and -5 (right) with involved sidechains are shown as stick model. (**B**) Protein structure overlay of TRX-f1 with TRX-m1 (gray and yellow, both models based on 2PU9) and 2-CysPRX (PDB: 5ZTE, green) revealed that TRX fold and nucleophilic Cys residues (represented as sticks) structurally align within less than 5Å root-mean-square deviation of atomic positions. Suggested 12-OPDA binding site is highlighted. (**C**) Free thiols of untreated (2-CysPRX and TRX-m1), reduced (2-CysPRXred and TRX-m1red) and reduced proteins treated with 12-OPDA at different ligand concentrations. Asterisks indicate significant difference (*p* ≤ 0.05) in comparison to control, calculated using one-way ANOVA with post hoc Tukey HSD.

Together with the fluorescent measurements, the results demonstrate that 12-OPDA interacts with TRX-f1 and covalently attaches to two Cys side chains of the thiol oxidoreductase. The two highest scoring binding poses performed with blind-docking tools SwissDock (www.swissdock.ch/docking, accessed on 28 January 2021) and CB-Dock (http://clab.labshare.cn/cb-dock/php/, accessed on 26 February 2021) of 12-OPDA to TRX-f1 suggested binding of 12-OPDA in proximity to fluorescence active tryptophan, tyrosine and DTNB-titratable Cys residues (Figure 6), supporting our experimental findings.

To confirm the covalent modification of TRX-f1 with 12-OPDA we performed an MS analysis of 12-OPDA in comparison with a mock-treated TRX-f1. 12-OPDA-treated TRX-f1 revealed an *m*/*z* increase of 586.411 ± 0.017, suggesting that two molecules of 12-OPDA (expected *m*/*z* = 292.204, found *m*/*z* 293.206 ± 0.009) added to TRX-f1 in line with the results of the DTNB assay (Figure 5A). The TRX-f1 construct (see Figure S4) had a predicted monoisotopic mass of 15472.981 which deviates from the experimentally found TRX-f1 mass. Our assumption is that the mass difference is due to methionyl loss, an often observed phenomenon [42], theoretically resulting in an *m*/*z* of 15341.940 which is highly similar to our observed mass of 15339.918 ± 0.002. For respective raw data and the summary of MS analysis, see Figure S5 and Table S1.

The above results suggest that the interaction of 2-cyclopentenone moiety-containing oxylipins with TRX is a phenomenon common to plants and animals. To address reaction specificity, we broadened the view to cover other chloroplast proteins with the TRX-fold present in various proteins harboring active site cysteinyls. To this end, we probed TRX-m1 and 2-CysPRX for interactions with 12-OPDA. TRX-m1 is expected to occur at similar concentrations compared to TRX-f1 whereas 2-CysPRX is perhaps the most abundant TRX-fold protein in planta ([43], Table 1; and Pax DB entries at https://pax-db.org/, accessed on 4 December 2020 [44]) with concentrations exceeding 100 µM. As can be seen in Figure 6B, 2-CysPRX and TRX-m1 both match structural features of TRX-f1 and their nucleophilic Cys residues align with less than 5Å deviation. A Trp residue is within 6Å close to the Cys residues of 2-CysPRX (see PDB: 5ZTE) and TRX-m1 has the WCGPCK motif, suggesting binding of 12-OPDA to both proteins in a similar mode as for TRX-f1. As revealed in Figure 6C, 12-OPDA decreased the thiol content of TRX-m1red and 2-CysPRXred. Significant thiol modification already occurred at ligand/protein ratio of 3.4 for TRX-m1 in comparison to 6.8 for 2-CysPRX. In the TRX-m1red sample, a 14-fold molar excess of 12-OPDA modified approximately one thiol group; however, 12-OPDA was unable to modify the reduced proteins at the two Cys sites, as observed for TRX-f1 (Figure 5A). With similar protein concentrations as employed for the DTNB assay and tested ligand/protein ratios of 18.4, an impact on the intrinsic fluorescence (both in λ_Em-max_ and fluorescence intensity) irrespective of 2-CysPRX redox state was evident (Table 2, Figure S3). At non-physiological TRX-f1 and TRX-m1 concentrations of 112 µM, 12-OPDA caused a red shift of the emission maxima of 2-CysPRX (36 µM) by 0.5 ± 0.0 nm, albeit no change in intensity occurred as observed for TRX-f1 (36 µM) and TRX-m1 (36 µM) (Figure S6).

These combined data indicate that 2-CysPRX binds to 12-OPDA at sites distinct from the suggested cavity in Figure 6B.

Activity tests revealed that the 2-CysPRX ability to scavenge H_2_O_2_ in the presence of NADPH thioredoxin reductase C (NTRC) was impaired by 12-OPDA, MVK and CP, but not by CPa (Figure S7), suggesting that CCCs also affect 2-CysPRX activity or interact with NTRC.

### 3.3. The Interaction of 12-OPDA with Cyclophilin 20-3

2-CysPRX and TRX-m1 physically interact with Cyp20-3 and regulate Cyp20-3 activity in response to redox alterations [32,33,45,46]. This type of reversible activity regulation involves Cys sidechains of Cyp20-3. The thiol redox state affects conformation and peptidyl-prolyl-*cis*/*trans*-isomerase activity of Cyp20-3 [32]. Cyp20-3 interacts with the polyunsaturated fatty acids-derived CCCs 12-OPDA (this study, [7,19]) and 4-hydroxynonenal (4-HNE) [10]. 4-HNE is a molecule with similar thiol reactivity as MVK [47]. To provide a first insight into relative binding affinities of CCCs to Cyp20-3, we conducted protein-fluorescence studies similar to the protocol mentioned in [19]. As seen in Table 3, MVK and 12-OPDA altered intrinsic fluorescence intensity of Cyp20-3, while addition of CP and CPa to Cyp20-3 did not cause any significant change in fluorescence (see Table 3 and Figure S8).

To test whether 12-OPDA and MVK covalently attach to free thiols of Cyp20-3, a DTNB assay was conducted. Although reduced Cyp20-3 contains four cysteines, only one reactive thiol (1.19 ± 0.4 mol mol^−1^, n = 8) was experimentally found. The sample Cyp20-3 (20 µM) treated with 300 µM MVK contained almost no free thiols (0.34 ± 0.03 mol mol^−1^, n = 8, *p* ≤ 0.05), while the addition of 300 µM 12-OPDA led to no significant change in thiol content (1.14 ± 0.3 mol mol^−1^, n = 8) in comparison to untreated reduced Cyp20-3. Modeling approaches (Figures S9 and S10) revealed that the distance between 12-OPDA or MVK to the C129, which is located in the vicinity of the RWFH-motif essential for PPIase activity, exceeds 6 Å, i.e., they are beyond the contact distance. In contrast to 12-OPDA, MVK was found in interaction clusters of 5–6 Å to cysteinyl residues (SwissDock result Figure S9) suggesting higher affinity of MVK to Cyp20-3 cysteinyls in support of the DTNB results. Like 12-OPDA, CP, CPa and JA did not reveal any contact sites to Cys thiols of Cyp20-3. The highest scoring hit of 12-OPDA-Cyp20-3 docking models involved the active site amino acid motifs R69, W135, F74, H140 (see Figures S9 and S10 and Table S2). The unbiased molecular docking results support our experimental findings obtained from intrinsic fluorescence quenching (Table 3) namely that among the tested ligands 12-OPDA quenched strongest. Furthermore, the combined results showed that Cyp20-3 is a non-covalent interaction target of 12-OPDA independent of thiols of Cyp20-3.

**Figure 7:**
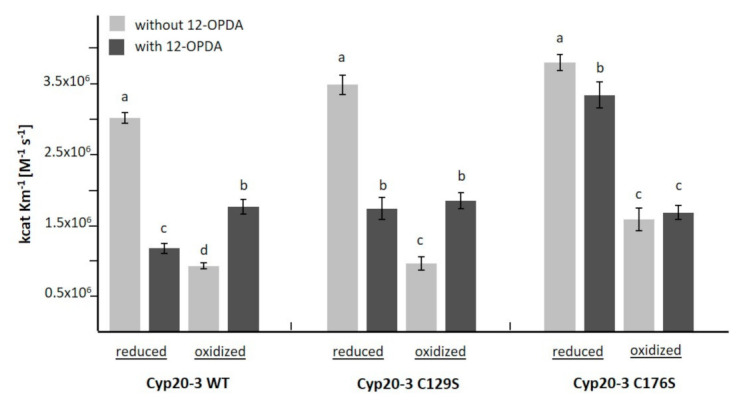
Activity of oxidized and reduced Cyp20-3 in the presence of 12-OPDA. Changed catalytic efficiency of PPI activity of Cyp20-3 validated the interaction with 12-OPDA. While reduced protein was inhibited by 12-OPDA, activity of oxidized protein increased. The activity of mutated C176S was only minimally affected by 12-OPDA. Data are means ± SD, n > 5. Significant differences at *p* ≤ 0.05 were calculated using one-way ANOVA with post hoc Tukey HSD and are represented by letters a–d.

The next experiments addressed the question of whether the interaction with 12-OPDA influences the catalytic efficiency of PPIase activity of Cyp20-3. Recombinant Cyp20-3 was oxidized or reduced, incubated with 12-OPDA for 10 min and the catalytic efficiency was determined. In accordance with previous studies [32], reduction of Cyp20-3 led to a significant increase in PPI activity, while oxidation inhibited the activity (see Figure 7). Incubation with 12-OPDA decreased the activity of reduced Cyp20-3 WT. The inhibitory effect was slightly less in the C129S variant. On the other hand, 12-OPDA stimulated the activity of oxidized Cyp20-3 WT and C129S to a similar extent. The sensitivity of catalytic PPIase efficiency to 12-OPDA was abolished in the oxidized C176S variant and greatly decreased in the reduced form, indicating the involvement of C176 in the interaction with 12-OPDA.

No activity measurements could be performed with Cyp 20-3 C171S and Cyp 20-3 C54S as described in Section 2.14. As an alternate readout, we performed ITC studies in order to assess whether Cyp20-3 binding affinities and thermodynamics of interaction with 12-OPDA are affected when Cys residue 171 was mutated to Ser. As seen in Figure S11, both WT and C171S interacted with 12-OPDA. However, the derived binding parameters can only be interpreted with caution, due to the poor fit (5 × 10^4^ > χ^2^) of our final binding curves based on any given binding model. By application of a single binding site fit (ITC Microcal), the following data were obtained for Cyp20-3 C171S (∆H = −3.5 ± 1.4 kcal mol^−1^, n = 0.9 ± 0.3, K_D_ = 26 ± 8 µM) and Cyp 20-3 WT (ΔH = -2.0 ± 1.1 kcal mol^−1^, n = 1.3 ± 0.5, K_D_ = 35.7 ± 9 µM).

## 4. Discussion

A double bond conjugated to a carbonyl group generates a particular electrophilicity at the β-carbon of the double bond with high reactivity. Many studies have explored the properties of such reactive electrophilic species (RES) also with respect to their significance for cell biology [10,15,48,49]; however, reactivity comparisons with 12-OPDA are rare also because of the unavailability of 12-OPDA at reasonable costs and amounts. The recent establishment of a one-pot synthesis [19] and its up-scaling by Löwe et al. [50] offered new experimental opportunities due to better availability of 12-OPDA. The chemical similarity of certain prostaglandins in animals to 12-OPDA is intriguing and, therefore, TRX as identified target of prostaglandin in animals appeared interesting for our study [9]. In addition to comparing the reactivity of different CCCs, this study also aimed to deepen our understanding of the role of Cyp20-3 as 12-OPDA receptor [10,15,48,49].

The determined thiol depletion of GSH caused by 12-OPDA reached about 20% and was approximately half the efficiency observed by Dueckershoff et al. [14]. The difference may be explained by slightly different reaction conditions. In the light of pH-variation among different organelles [51] and particularly in chloroplasts during the light/dark cycle, it appears interesting that the reactivity of 12-OPDA with thiols was three-fold higher at pH 8.0 than at pH 6.6. This is particularly interesting if thiol-dependent redox protein–protein interactions are considered, see [33], as discussed below for the interaction of 12-OPDA with Cyp20-3 and TRX-fold proteins. At pH 7.2, 12-OPDA led to the same GSH decrease as HEX similar to the study by Davoine et al. [1], while at pH 8.0 12-OPDA reacted stronger with GSH than HEX. It is striking to note that CP reacted stronger with GSH than 12-OPDA at all pH values (Figure 1). This is probably due to the positive inductive effect of substituents at the 12-OPDA ring, lowering the positive partial charge at the β-carbon of the ’ene’ moiety. Thus, the substitution pattern opposite to the CP ring double bond likely influences the reactivity of CCC with GSH. For this reason, it would be interesting to include phytoprostanes and dn-OPDA in the comparison.

The observation that the 12-OPDA-Et retained its reactivity toward GSH similar to 12-OPDA is of significant interest for future plant research and application since the presumed higher membrane permeability of the ethylester likely eases its administration to intact plant tissue. The hydrophobicity of 12-OPDA-Et is 15% higher than that of 12-OPDA as calculated by the logP value (see Table 1).

Cys reacts more strongly with CCCs than GSH [2]. Therefore, the observed conjugation of 12-OPDA with Cys was expected (Figure 4). However, its reaction with ABA was less expected since conjugation between GSH and ABA was undetectable (Figure 1). Nevertheless, ABA formed an adduct with Cys (Figure 2) of yet uncharacterized molecular structure. This is similar to observations on thiol adduct formation with β-substituted α,β-cyclohexenone derivatives [52]. The reactivity described here points to possible physiological interactions of ABA with thiols or other sulfur-containing biomolecules and should be further explored. The identified interaction of 12-OPDA with cysteinyl thiols (Figure 4) was experimentally expanded to the protein level since many proteins display exposed cysteinyl side chains.

The described interaction of prostaglandins with animal TRX prompted us to investigate a possible interaction of 12-OPDA with TRX-f1, TRX-m1, 2-CysPRX and Cyp20-3. 12-OPDA decreased TRX-f1 activity as shown by TRX-f1-dependent activation of FBPase, suggesting modification of the nucleophilic site of TRX-f1 as proposed by the modeling approach (Figure 6A). The adduct formation was validated by mass spectrometry and revealed a stoichiometry of 2 mol 12-OPDA per mol TRX-f1. The docking results suggest that two out of three cysteines, namely C99 (nucleophilic) and/or C102, might be targeted by 12-OPDA, similar as described by the two-fold modification of TRX by 15dPGJ_2_ [18]. The physiological relevance of the in vitro interaction between 12-OPDA and TRX-f1 shown in Figure 5C awaits elucidation. One hypothetical scenario derived from studies on mutant plants with altered FBPase activity [53] might be the following: Under stress conditions, when plants are in demand of defending themselves against the impact of stress, energy consumption in the Calvin–Benson cycle needs to be decreased. Stress-induced synthesis of 12-OPDA might participate in downregulation of chloroplast TRX-f1-dependent FBPase activity, redirecting carbon flux from starch synthesis and regeneration of ribulose-1,5-bisphosphate to export and allowing for energization of the cytosol. In context of sugar and redox homeostasis, 12-OPDA might also affect NADPH fluxes especially through generation of NADPH via inhibiting the reductive regeneration of TRX-f1 or TRX-m1. Further complexity in 12-OPDA function in vivo arises from dark/light effects on metabolism [30], regulation of RES and ROS defense-genes by 12-OPDA [14] and the glutathionylation of TRX-f1 or TRX isoforms by oxidized GSH [54].

Under heat- and high light-stress, 12-OPDA binds to Cyp20-3 and stimulates synthesis of O-acetyl serine, the precursor of Cys synthesis, by activating the thiol redox-sensitive cysteine synthase complex in chloroplasts [7]. The involved regulatory COPS module consists of cyclophilin 20-3 (Cyp20-3), O-acetylserine (thiol)lyase B (OASTL-B), 2-cysteine peroxiredoxins A/B (2-CysPRX) and serine acetyltransferase 2;1 (SERAT2;1) [55]. The binding of 12-OPDA regulates this module; however, data were lacking as to whether binding of 12-OPDA to Cyp20-3 is reversible or involves the reactivity as CCC, and whether OPDA interferes with the PPIase activity of Cyp20-3. As seen in Figure 7, 12-OPDA inhibited the PPIase activity of reduced Cyp20-3. Our modeling approaches (Figures S9 and S10) suggest that the interaction between 12-OPDA and Cyp20-3 proceeds in proximity to the PPIase active site (RWFH motif) similar as shown for the human Cyp and Cyp20-3 inhibitor cyclosporin A (CsA) [56]. In context of plant infections 12-OPDA might act as a CsA-like effector or as competitor for unidentified Cyp20-3 ligands derived from pathogens. The effect of 12-OPDA on the interaction of Cyp 20-3 with VirD2 or in the interaction of human Cyp with viral proteins is worth being investigated as this topic has not been addressed so far (see [57,58]). It is intriguing that PPIase activity of oxidized Cyp20-3 was stimulated by 12-OPDA. We are not aware of similar observations where electrophiles activate oxidized thiol-dependent redox biomolecules and we propose that 12-OPDA enhances substrate binding by shaping the conformation of the active site in the oxidized Cyp20-3.

The change in catalytic efficiency of PPIase activity of Cyp20-3 and its variants under reducing and oxidizing conditions in the presence of 12-OPDA (Figure 7) showed that 12-OPDA dynamically affects the conformation and activity of Cyp20-3. These data go beyond the findings of Park et al. [7] who reported a Cyp20-3-dependent increase of chloroplast thiol content under high light or heat shock. The protective effect of 12-OPDA during a heat shock includes upregulation of chaperones [14,59]. The result that Cyp20-3 Cys variants respond differently to 12-OPDA might indicate an environmental fine-tuning of the multiple functions of Cyp20-3 by 12-OPDA. The entire network awaits elucidation, because the redox interaction partners of Cyp20-3, TRX-m1 and 2-CysPRX also reacted with 12-OPDA (Figure 6C, Table 2 and Supplementary Materials). As shown for the in vitro interaction between 2-CysPRX and NTRC, the reactivation of 2-CysPRX by NTRC in the presence of 42.5 µM 12-OPDA was impaired. Whether NTRC might also be modified by 12-OPDA was not addressed herein as we felt this topic was beyond the scope of our work. It remains to be investigated whether two domain proteins such as NTRC or the EF-hand-TRX fold protein calredoxin [60] interact with CCCs.

Recently, Cheong et al. [45] demonstrated that the interaction of Cyp20-3 with TRX-m1 depends on the 12-OPDA concentration, supporting our observation that TRX-m1 is sensitive towards 12-OPDA. The 12-OPDA sensitive NTRC-2-CysPRX redox module (see Figure S7) adds to the 12-OPDA sensitive protein-protein interaction elucidated in [45]. Mano et al. [49] searched for proteins subjected to 4-HNE-modification and identified Cyp20-3 and cysteine synthase among this group. In addition, homologues of Cyp20-3 in rat, yeast and humans (Uniprot IDs: P10111, P14832, P62937) were also found to be modified by RES [38,48]. The literature data support the affinity of Cyp20-3 towards CCCs as observed here for 12-OPDA and MVK (Table 3). Of the tested compounds, MVK was capable of modifying one of the Cyp20-3 thiols. This was similar to the observation described in [38] showing a single thiol modification in human Cyp. The Cyp20-3 modeling approach with 12-OPDA and MVK revealed that the only Cys that was found in close vicinity to the docked ligands was C176 and this interaction only occurred for MVK performed with SwissDock (Figure S9). However, docking results are predictions and the noted insensitivity of the C176S variant to added 12-OPDA (see Figure 7) hints to an interaction site of 12-OPDA next to the hypothesized PPIase-RWFH motif in Cyp20-3. This could be stabilized when C171 is absent and fails to form a disulfide bridge with C54, as the Cyp 20-3 C171S mutant did not seem to be impaired in associating with 12-OPDA (see Figure S11). Interestingly, C176 distinguishes Cyp20-3 from all cyclophilins in *A. thaliana* [32]. Crystallographic studies are required to resolve Cyp20-3s ligand interaction site(s).

## 5. Conclusions

This report provides novel insight into covalent and noncovalent interactions of 12-OPDA and related CCCs with thiol-containing biomolecules. In addition to the formation of adducts with cysteine, including a novel reaction product between cysteine and abscisic acid, the results demonstrate the potential role of 12-OPDA in tuning protein function within the cellular redox regulatory network. Similar to cyclopentenone moiety-containing prostaglandins which react with human TRX, 12-OPDA modified plant TRXs. Thus, two molecules of 12-OPDA covalently bound to TRX-f in vitro and inhibited its ability to activate oxidized inactive FBPase. Thus, OPDAylation might have physiological significance which should be explored in future research. In a converse manner, 12-OPDA acted as reversible non-covalent effector of CYP20-3 which modified its PPIase activity. The search for possibly OPDAylated targets ex vivo should concentrate on chloroplast proteins, since 12-OPDA is synthesized in the stroma, while 12-OPDA likely is rapidly reduced once exported from the plastids.

## Figures and Tables

**Table 1:** Properties of 12-OPDA and related compounds. The list gives chemical properties, common names and predicted physicochemical and pharmacological properties of conjugated carbonyl compounds (CCCs) and related compounds. TPSA, topological polar surface area; logP, partition coefficient; RBN, number of rotatable bonds. The table also provides some information on bioactivities of the compounds as given in the text.

Sum Formula	Name	Charge at pH 7	RBN	logP	TPSA	Physiological Relevance
C_18_H_28_O_3_	12-Oxophytodienoic acid, 12-OPDA	−1	11	4.58	54.37	2-cyclopentenone prostaglandin analogue, thiol metabolism, stress response, JA precursor [9]
C_20_H_32_O_3_	12-OPDA-ethylester, 12-OPDA-Et	0	11	5.27	43.38	Ethylester of 12-OPDA, unknown
C_5_H_6_O	2-Cyclopentenone, CP	0	0	0.40	17.07	Structural subunit of 12-OPDA and other CP-derivatives
C_4_H_6_O	Methyl vinyl ketone, MVK	0	1	0.50	17.07	Isoprene and lipid-derived stress-related oxidation product [4,5]
C_5_H_8_O	Cyclopentanone, CPa	0	0	0.89	17.07	Structural subunit of MJ and various PGs
C_6_H_10_O	Trans-2-hexenal, HEX	0	3	2.33	17.07	Green leaf volatile, stress response [3]
C_9_H_16_O_2_	4-Hydroxynonenal, 4-HNE	0	6	2.46	37.30	Lipid-derived stress-related oxidation product [10]
C_15_H_20_O_4_	Abscisic acid, ABA	−1	3	2.10	74.60	Isoprenoid-derived 2-cyclohexenone compound, sulfur and thiol metabolism, leaf abscission, stress response, germination [6,8]
C_20_H_28_O_3_	15-Deoxy-Δ12,14-prostaglandin J2, 15dPGJ_2_	−1	11	5.21	54.37	Mammalian 2-cyclopentenone prostaglandin, thiol metabolism, stress response [11,12]
C_20_H_30_O_4_	Prostaglandin J2, PGJ_2_	−1	12	3.76	74.60	Mammalian 2-cyclopentenone prostaglandin, thiol metabolism, stress response [12]

**Table 2:** Analysis of interaction between TRX-fold proteins and 12-OPDA via protein fluorescence. Data are from experiments similar to the one presented for TRX-f1red, untreated TRX-f1_,_ TRX-m1red_,_ untreated TRX-m1, 2CysPRXred_,_ untreated 2CysPRX ±12-OPDA in Figure S3. Results are presented as means ± SD, n is given in the last column. Asterisks indicate significant differences at *p* ≤ 0.05 in comparison to samples incubated with plain solvent. The fluorescence intensity [r. U.] result of sample TRX-f1red + 12-OPDA at a ligand/protein ration of 9.6 in comparison to TRX-f1red showed a trend, but was insignificant (*p* = 0.11). Statistical evaluation employed one-way ANOVA with post-hoc Tukey HSD test. For details, see Section 2.

Sample	[ligand]/[protein]	λ_Em-max_ [nm]	Fluorescence intensity [r. U]	n
TRX-f1red	0	342.2 ± 0.3	73.3 ± 4.4	3
TRX-f1red + 12-OPDA	9.6	341.3 ± 0.4	64.6 ± 2.9	2
TRX-f1red + 12-OPDA	19.2	341.7 ± 0.3	60.1 ± 3.2 *	3
TRX-f1_untreated_	0	341.3 ± 0.8	82.2 ± 0.6	3
TRX-f1_untreated_ + 12-OPDA	18.4	342.2 ± 0.3	78.6 ± 1.4 *	3
TRX-m1red	0	327.0 ± 0.4	81.6 ± 1.8	4
TRX-m1red + 12-OPDA	18.4	328.5 ± 0.7	73.0 ± 1.4 *	4
TRX-m1_untreated_	0	315.3 ± 0.3	74.3 ± 2.2	4
TRX-m1_untreated_ + 12-OPDA	18.4	315.1 ± 0.5	68.6 ± 1.3 *	4
2-CysPRX_untreated_	0	338.3 ± 0.3	86.9 ± 0.9	6
2-CysPRX_untreated_ + 12-OPDA	18.4	339.3 ± 0.3 *	83.1 ± 0.6 *	4
2-CysPRXred	0	335.2 ± 0.3	97.1 ± 1.5	3
2-CysPRXred+12-OPDA	18.4	336.5 ± 0.4 *	92.5 ± 0.9 *	4

**Table 3:** Intrinsic protein fluorescence analysis of Cyp20-3 incubated with various CCCs. A total of 30 µM Cyp20-3 was mixed with indicated compounds at a final concentration of 125 µM in a quartz cuvette and analyzed for intrinsic fluorescence changes. Among the tested compounds, 12-OPDA and MVK changed the intrinsic fluorescence in comparison to the control. Data are means ± SD (n ≥ 6). Asterisks indicate significant differences at *p* ≤ 0.05. Statistical significance was calculated with one-way ANOVA with post-hoc Tukey HSD test. For details, see Section 2.

Additive	Change in Fluorescence Intensity [r. U] at λ_Em-max_ [nm] Relative to Control	λ_Em-max_ [nm]	
		Treatment	Control
12-OPDA	−2.52 ± 0.50 *	331.63 ± 0.95	330.13 ± 0.25
MVK	−1.48 ± 0.48 *	330.80 ± 0.57	330.25 ± 0.87
CP	−0.66 ± 0.7	330.33 ± 0.29	330.00 ± 0.00
CPa	−0.24 ± 0.5	330.13 ± 0.25	330.38 ± 0.48

## Data Availability

All necessary data are contained in this paper.

## Supplementary Materials

The following data are available online at https://www.mdpi.com/2218-273X/11/3/457/s1, Table S1. Summary of analysis of TRX-f1-12-OPDA interaction; Table S2. Cyp20-3 top docking scores for several compounds computed on two online servers; Figure S1. Chemical structures of 12-OPDA and related compounds; Figure S2. ITC analysis of ABA and Cys; Figure S3. Influence of 12-OPDA on the intrinsic fluorescence emission spectra of TRX-fold proteins in untreated or reduced form; Figure S4. Protein sequence and molar weight of TRX-f1 construct used in this study; Figure S5. Intact protein analysis of TRX-f1-12-OPDA via mass spectrometry before and after incubation of TRX-f1 with 12-OPDA Figure S6. Interaction of 12-OPDA with reduced TRX-f1, TRX-m1 and 2-CysPRX at high protein concentrations analyzed by intrinsic protein fluorescence. Figure S7 NTRC-coupled peroxidase activity of 2-CysPRX in the presence of CCCs; Figure S8. Influence of 12-OPDA and related compounds on the intrinsic fluorescence emission spectra of Cyp 20-3; Figure S9. Docking study of Cyp20-3 with 12-OPDA and MVK performed with SwissDock; Figure S10. In silico interaction study of Cyp20-3 with 12-OPDA and MVK performed with CB-Dock; Figure S11. Calorimetric analysis of the 12-OPDA interaction with Cyp20-3 and Cyp20-3 C171S.
