# Supplementary material for: The In Vitro Interaction of 12-Oxophytodienoic Acid and Related Conjugated Carbonyl Compounds with Thiol Antioxidants
Source: Biomolecules. 2021 Mar 18;11(3):457. doi: 10.3390/biom11030457 (PMC8003295; doi:10.3390/biom11030457)
Supplement: Supplementary file 1 [file biomolecules-11-00457-s001.pdf]

biomolecules

MDPI

Supplementary Materials

The *in vitro* interaction of 12-oxophytodienoic acid and related conjugated carbonyl compounds with thiol antioxidants *by* Daniel Maynard, Andrea Viehhauser, Madita Knieper, Anna Dreyer, Ghamdan Manea, Wilena Telman, Falk Butter, Kamel Chibani, Renate Scheibe, Karl-Josef Dietz

Table S1. Summary of analysis of TRX-f1-12-OPDA interaction. Mass spectrometric analysis of 12-OPDA incubated with TRX-f1, data see Figure S5.

MS analysis mode	Mass of TRX-f1	Mass of TRX-f1+12-OPDA	Mass difference	Mass difference/2
none	15339.917205	15926.338917	586.421712	293.210856
CID20	15339.920423	15926.338293	586.417870	293.208935
CID30	15339.918796	15926.339694	586.420898	293.210449
CID40	15339.915995	15926.302113	586.386118	293.193059

Table S2. Cyp20-3 top docking scores for several compounds. The more negative the binding energy value (Gibbs free enthalpy), the more exothermic the proposed interaction. Full fitness and total scores of the online tool SwissDock indicate that lower values represent binding modes which are more favorable than higher scoring numbers. The best binding affinity scoring models of 12-OPDA, MVK, JA, CP, CPa docked to Cyp20-3 included contact (<6Å) to the RWFH motif of PPIase. Among these 12-OPDA showed most favorable interaction with Cyp20-3 followed by JA, CPa, CP, and MVK. First hit docking scores of CB-Dock (Vina score) are also shown. In line with SwissDock binding energies a lower Vina score is related to stronger binding tendency. All CB-Dock estimated binding cavities are shown for MVK and 12-OPDA in Figure S10.

	***In silico* tool**			
	**SWISS DOCK**			**CB-DOCK**
**Name**	**Full fitness (kcal mol^{-1})**	**Binding energy (kcal mol^{-1})**	**Total score**	**Vina Score (kcal mol^{-1})**
12-OPDA	-976.4	-8.5	-36.8	-5.1
JA	-966.4	-6.9	-19.3	-5.0
MVK	-921.0	-5.3	-9.3	-3.3
CP	-929.9	-5.6	-15.2	-3.9
CPa	-932.0	-5.4	-15.3	-4.0

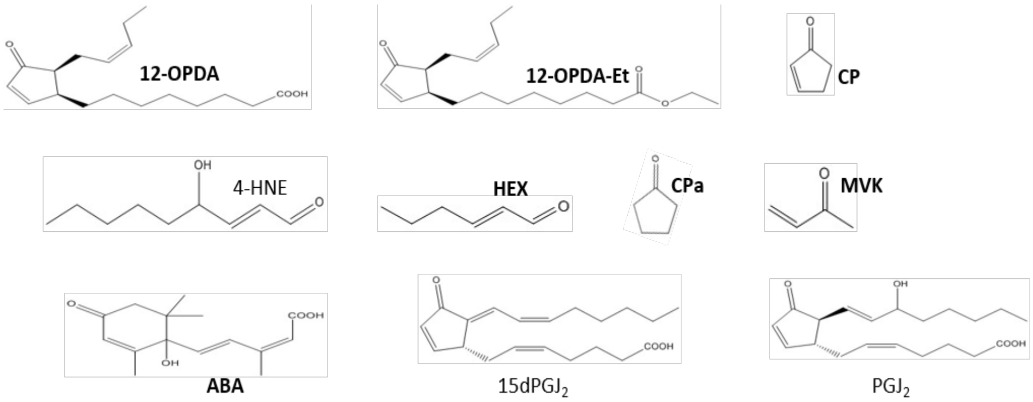

Figure S1. Chemical structures of 12-OPDA and related compounds. Compounds experimentally addressed herein are highlighted in bold. Details see text.

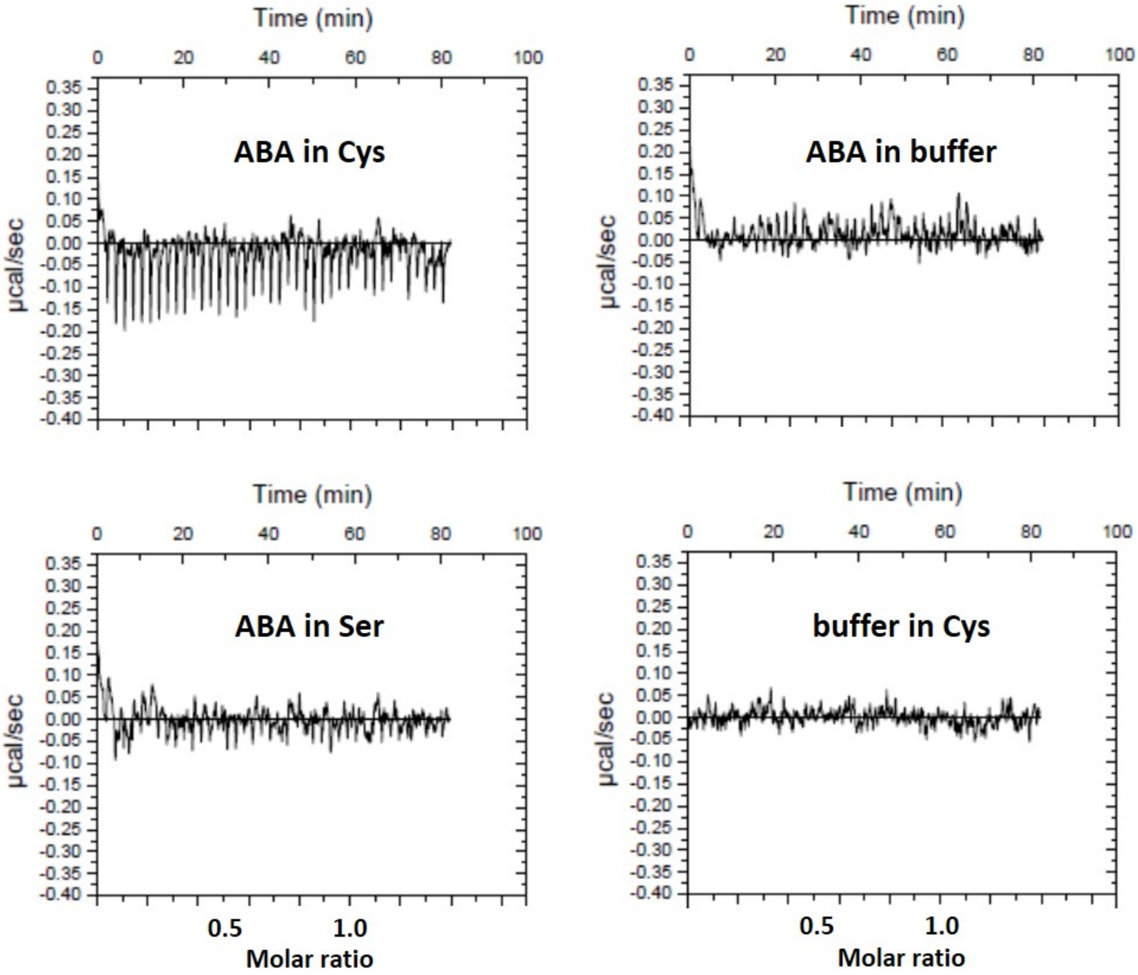

Figure S2. ITC analysis of the interaction between ABA and cysteine. Results are shown as differential response vs. molar ratio and time. ABA injected into cysteine (Cys) resulted in an exothermic response in comparison to the injection of ABA into serine (Ser), ABA into buffer, or Cys into buffer. Each titration, consisted of 40 injections of 5 µL 5 mM ABA or buffer (40 mM K-P$_i$, pH 8.0) injectant into cuvette filled with 1.3 mL 500 µM aminoacids or buffer was performed in triplicate with identical results. Molar ratios are defined as injectant/compound in the cuvette, per default the concentrations in the syringe and initial concentrations in the cuvette are set as 5 mM and 500 µM irrespective of content.

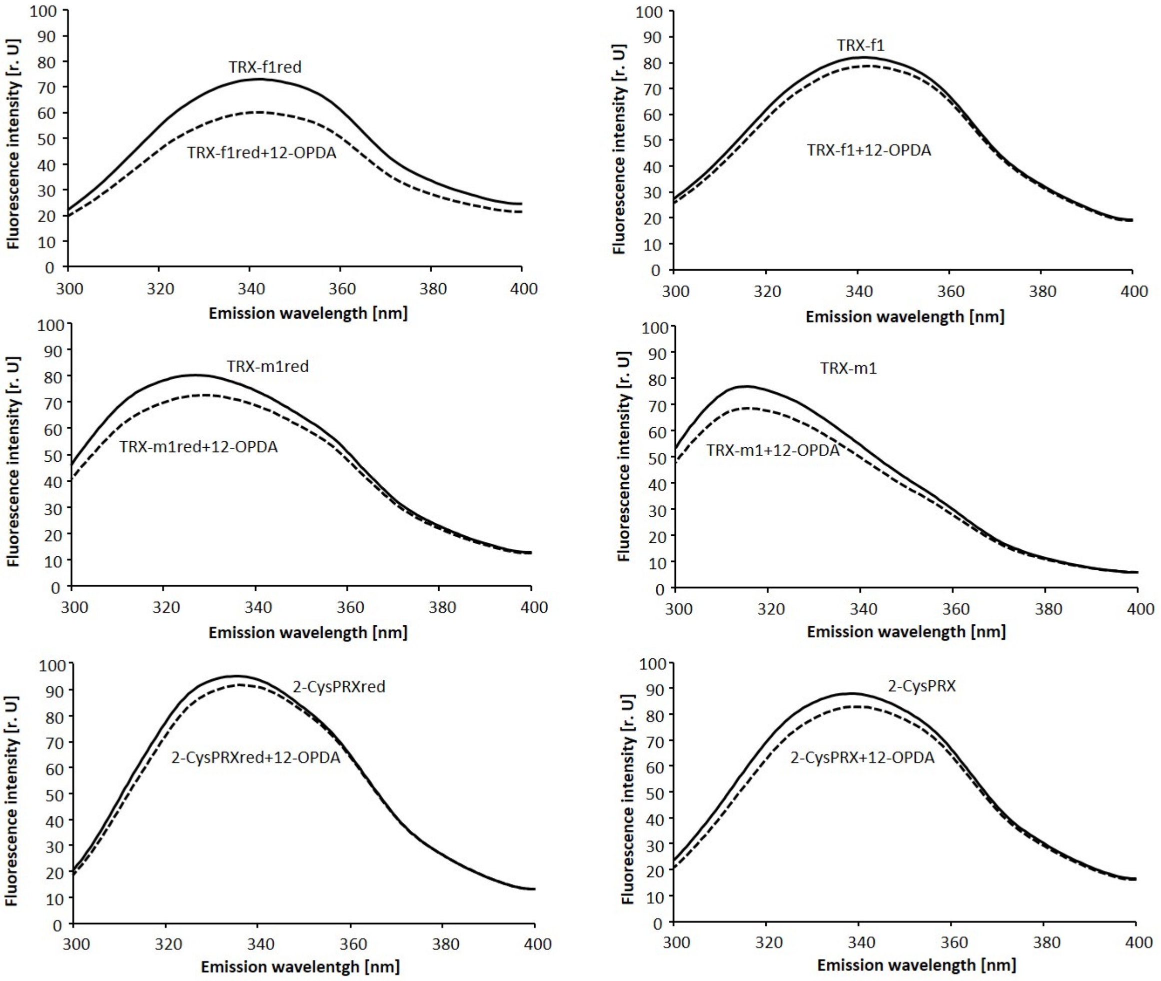

Figure S3. Influence of 12-OPDA on the intrinsic fluorescence emission spectra of TRX-fold proteins in reduced (left) or untreated (right) form. All proteins except TRX-f1 red were incubated at a ligand/protein ratio of 18.4 (the 12-OPDA/TRX-f1red ratio was 19.2) and measured as described in Materials and Methods. Experiments were performed at least three times, with identical results**.** Values see Table 2.

MGSSHHHHHHSSGLVPRGSHMSLETVNVSVGQVTEVDKDTFWPIVK
AAGEKLVVLDMYTQWCGPCKVIAPKYKALSEKYDDVVFLKLDCNPDN
RPLAKELGIRVVPTFKILKDNKVVKEVTGAKYDDLVAAIETARSAASG.
MW: 15472.98079

Figure S4. Protein sequence and molecular weight (MW) of TRX-f1 construct used in this study.

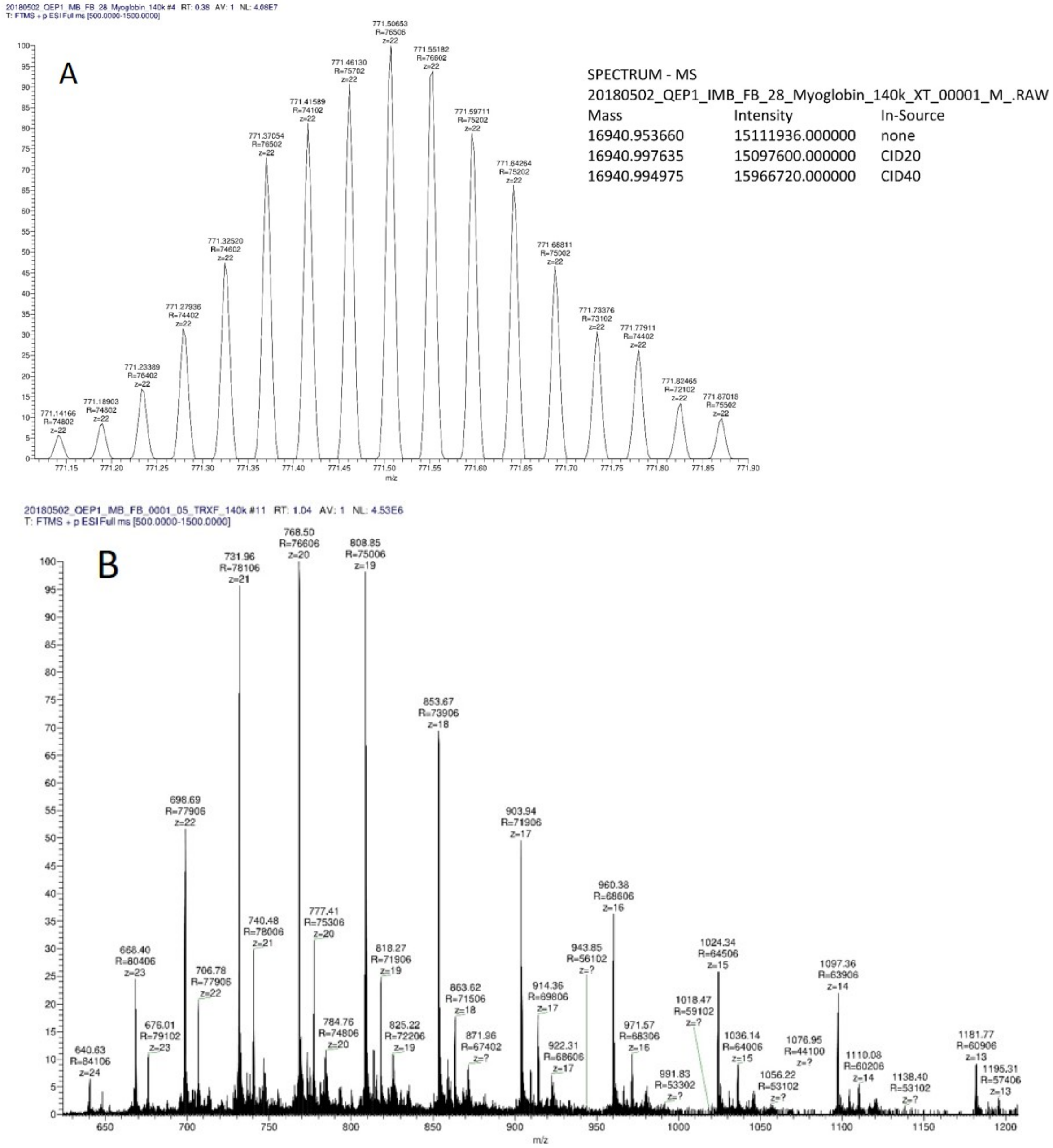
20180502_QEP1_IMB_FB_28_Myoglobin_140k #4 RT: 0.38 AV: 1 NL: 4.08E7
T: FTMS + p ESI Full ms [500.0000-1500.0000]
A
SPECTRUM - MS
20180502_QEP1_IMB_FB_28_Myoglobin_140k_XT_00001_M_.RAW
Mass Intensity In-Source
16940.953660 15111936.000000 none
16940.997635 15097600.000000 CID20
16940.994975 15966720.000000 CID40
m/z
20180502_QEP1_IMB_FB_0001_05_TRXF_140k #11 RT: 1.04 AV: 1 NL: 4.53E6
T: FTMS + p ESI Full ms [500.0000-1500.0000]
B
m/z

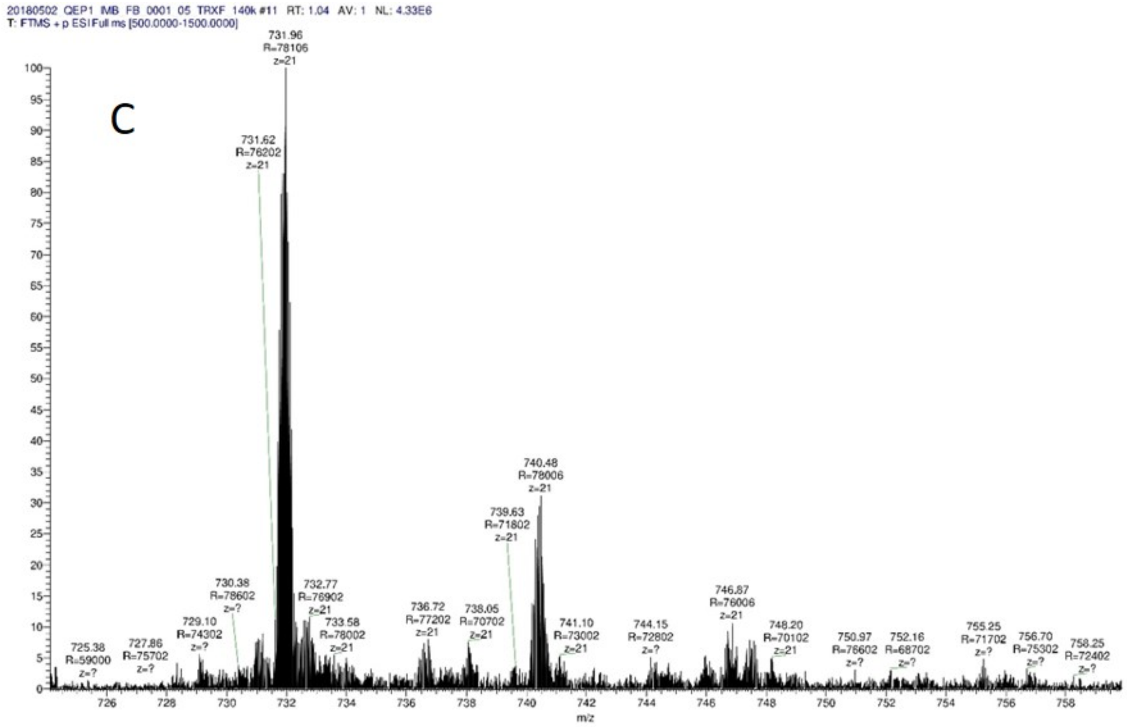
20180502_QEP1_IMB_FB_0001_05_TRXF_140k #11 RT: 1.04 AV: 1 NL: 4.33E6
T: FTMS + p ESI Full ms [500.0000-1500.0000]
C
731.96
R=78106
z=21
731.62
R=76202
z=21
740.48
R=78006
z=21
739.63
R=71802
z=21
746.87
R=76006
z=21
m/z

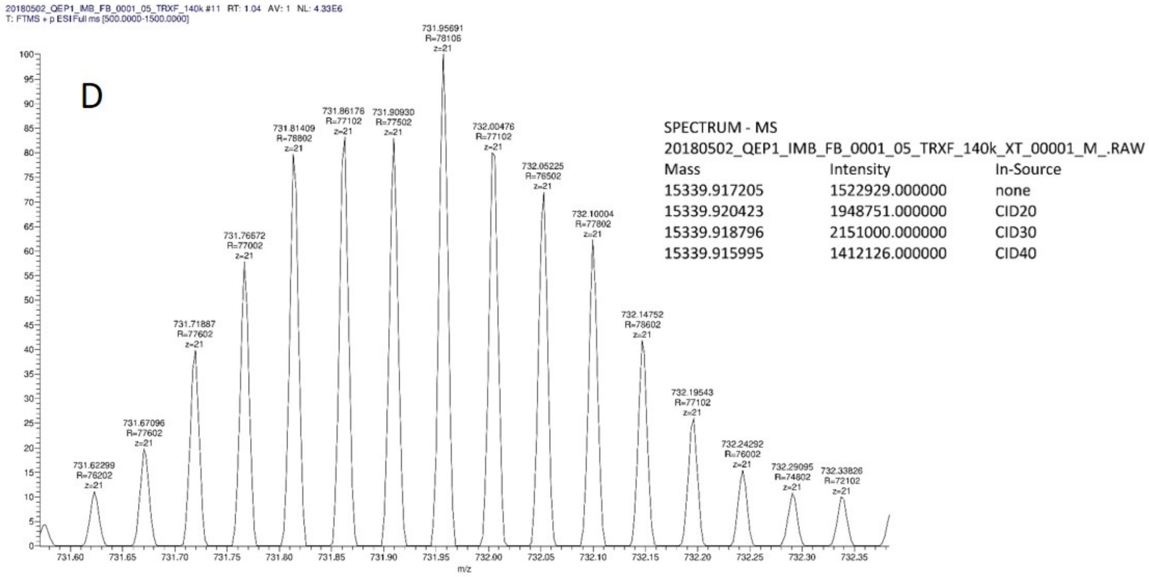
20180502_QEP1_IMB_FB_0001_05_TRXF_140k #11 RT: 1.04 AV: 1 NL: 4.33E6
T: FTMS + p ESI Full ms [500.0000-1500.0000]
D
731.95691
R=78106
z=21
SPECTRUM - MS
20180502_QEP1_IMB_FB_0001_05_TRXF_140k_XT_00001_M_.RAW
Mass Intensity In-Source
15339.917205 1522929.000000 none
15339.920423 1948751.000000 CID20
15339.918796 2151000.000000 CID30
15339.915995 1412126.000000 CID40
m/z

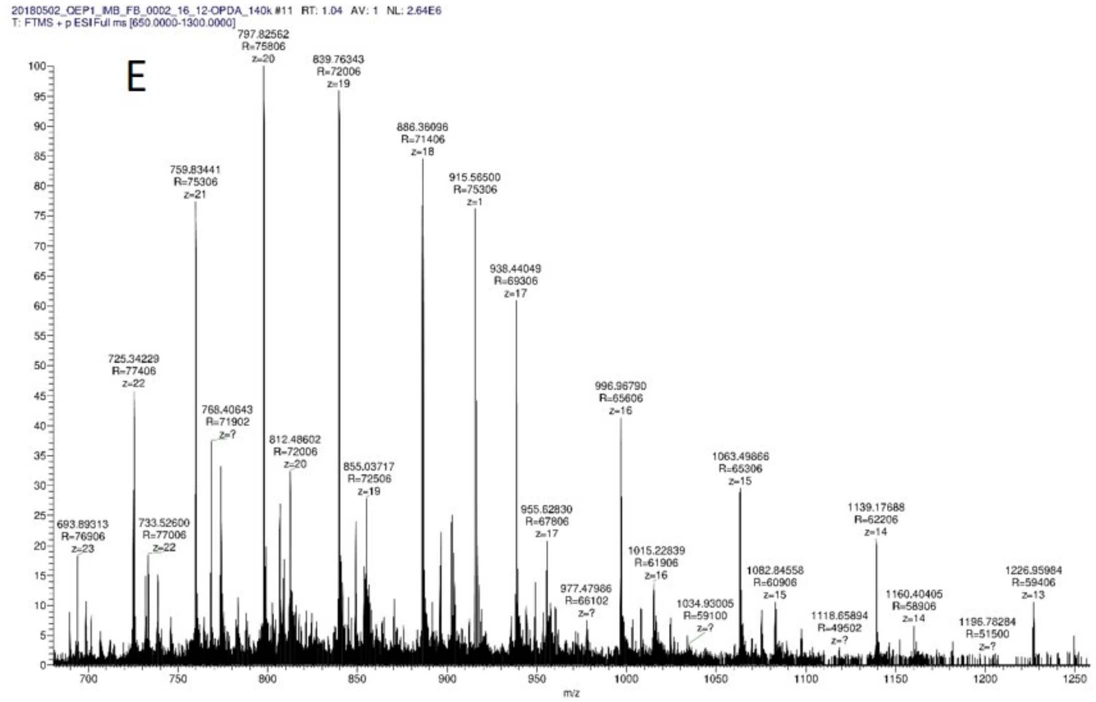
20180502_QEP1_IMB_FB_0002_16_12-OPDA_140k #11 RT: 1.04 AV: 1 NL: 2.64E6
T: FTMS + p ESI Full ms [650.0000-1300.0000]
E
797.82562 R=75806 z=20
839.76343 R=72006 z=19
886.36096 R=71406 z=18
915.56500 R=75306 z=1
759.83441 R=75306 z=21
938.44049 R=69306 z=17
725.34229 R=77406 z=22
996.96790 R=65606 z=16
768.40643 R=71902 z=?
812.48602 R=72006 z=20
855.03717 R=72506 z=19
1063.49866 R=65306 z=15
955.62830 R=67806 z=17
1139.17688 R=62206 z=14
693.89313 R=76906 z=23
733.52600 R=77006 z=22
1015.22839 R=61906 z=16
1082.84558 R=60906 z=15
1226.95984 R=59406 z=13
977.47986 R=66102 z=?
1034.93005 R=59100 z=?
1160.40405 R=58906 z=14
1118.65894 R=49502 z=?
1196.78284 R=51500 z=?
m/z

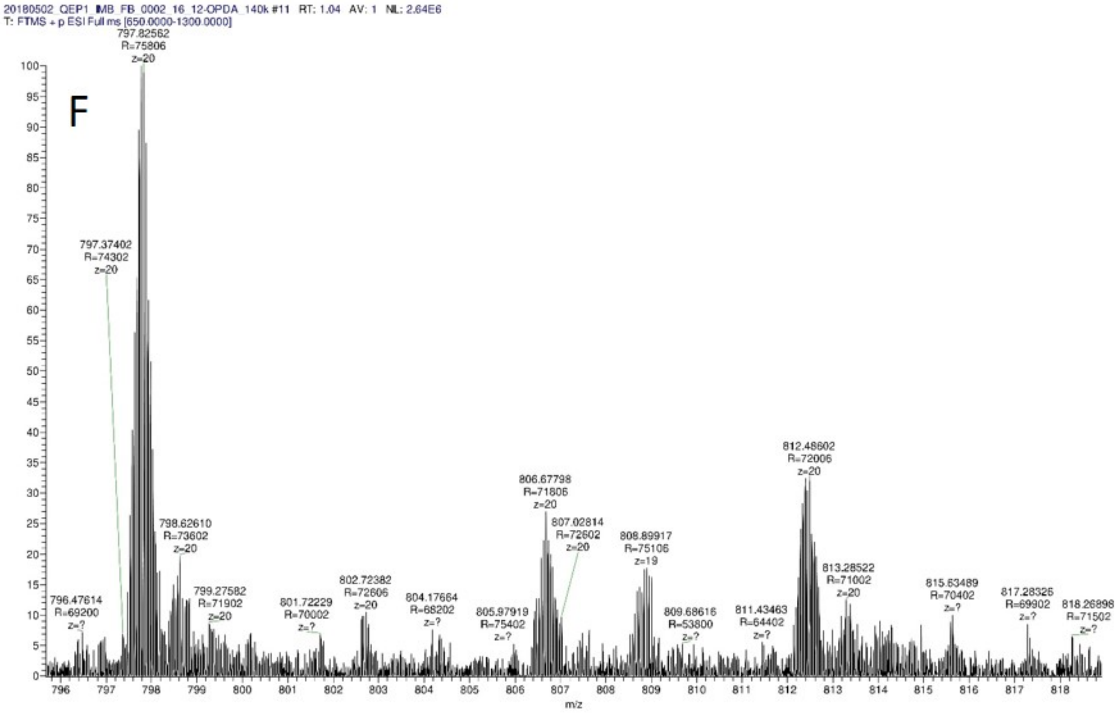
20180502_QEP1_IMB_FB_0002_16_12-OPDA_140k #11 RT: 1.04 AV: 1 NL: 2.64E6
T: FTMS + p ESI Full ms [650.0000-1300.0000]
F
797.82562 R=75806 z=20
797.37402 R=74302 z=20
812.48602 R=72006 z=20
806.67798 R=71806 z=20
807.02814 R=72602 z=20
798.62610 R=73602 z=20
808.89917 R=75106 z=19
813.28522 R=71002 z=20
799.27582 R=71902 z=20
802.72382 R=72606 z=20
796.47614 R=69200 z=?
801.72229 R=70002 z=?
804.17664 R=68202 z=?
805.97919 R=75402 z=?
809.68616 R=53800 z=?
811.43463 R=64402 z=?
815.63489 R=70402 z=?
817.28326 R=69902 z=?
818.26898 R=71502 z=?
m/z

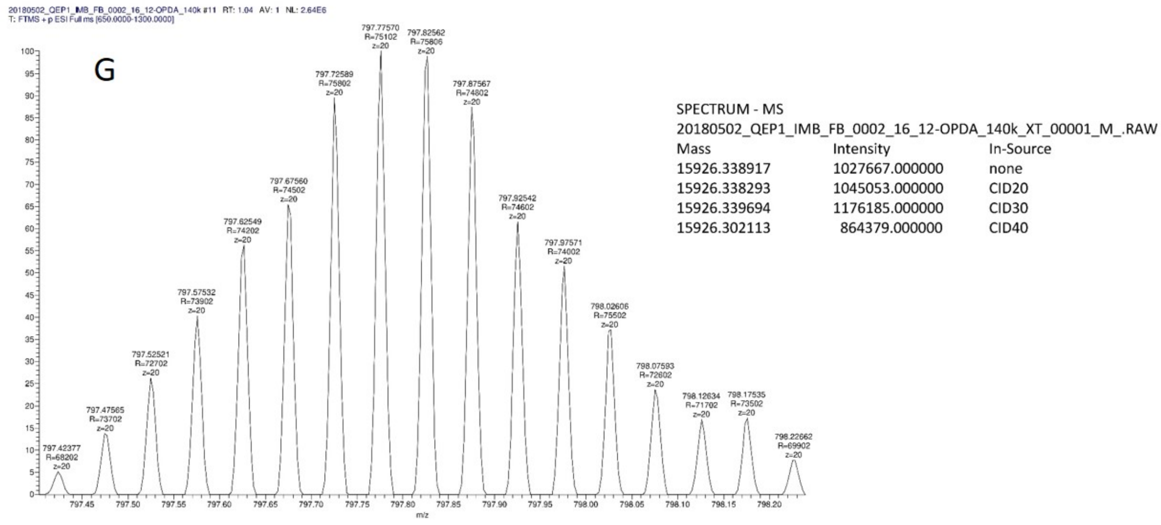

Figure S5. Intact protein analysis of TRX-f1-12-OPDA via mass spectrometry before and after incubation of TRX-fl with 12-OPDA. (**A**) Calibration of MS with myoglobin 5 pmol μL^{-1}, 140,000 resolution shows protein peaks with isotope distribution for charge state 22+. (**B**)-(**D**) Spectra of TRX-f1, in (B) overview of the full m/z range with different charge states, (C) focus on charge state 21+ and (D) isotope distribution of charge state 21+ used for mass determination. **E-G** spectra of TRX-f1 incubated with 12-OPDA, in (E) full m/z range with different charge states, (F) zoom of m/z window for charge state 20+ and (G) isotope distribution of charge state 20+ used for mass determination. Results after using Xtract (Thermo) deconvolution and deisotoping on our isotopically resolved protein mass spectra from (**D**) and (**G**) both 30 ng μL^{-1}, 140,000 resolution are shown in insets. All samples were injected at a rate of 20 μL min^{-1} (ESI-Source). Further Details see Materials and Methods.

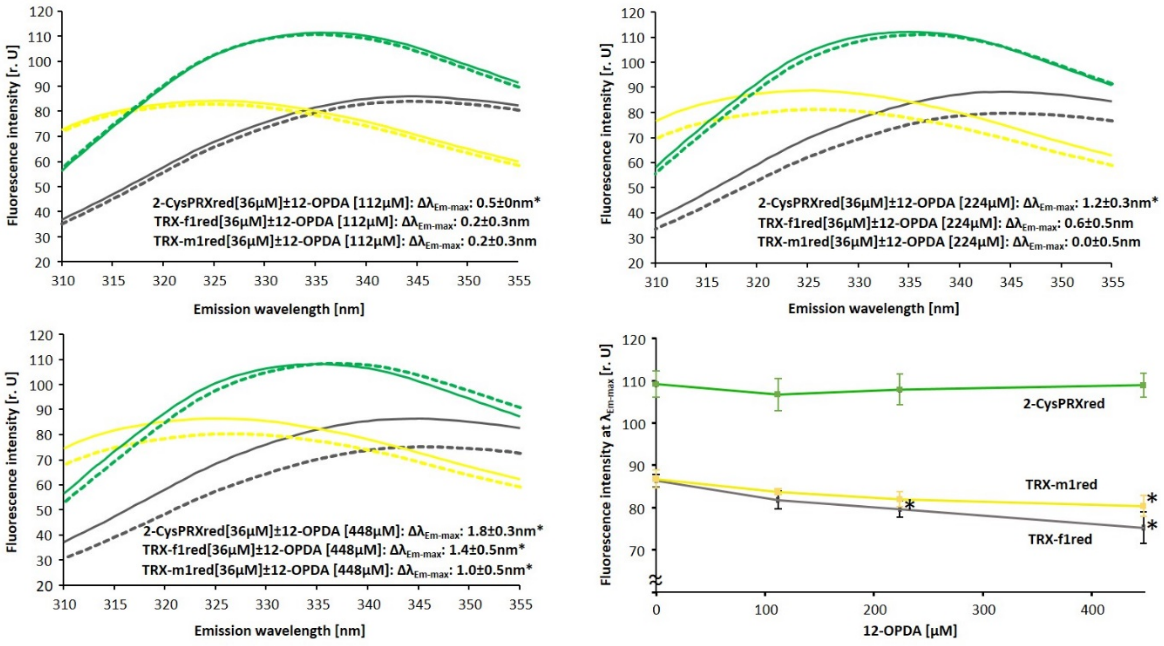

Figure S6 Interaction of 12-OPDA with reduced TRX-f1, TRX-m1 and 2-CysPRX at high protein concentrations analyzed by intrinsic protein fluorescence. Reduced and desalted proteins (36 μM) were incubated with 12-OPDA at indicated concentrations (Upper left: 112 μM, upper right: 224 μM, lower left: 448 μM) and analyzed as described in Materials and Methods. The emission spectra (broken line: +12-OPDA, solid: control=0 μM 12-OPDA) are representative of one measurement. All experiments were repeated at least three times with identical results, as seen in insets denoting changes (mean ± SD of n≥3) of emission maxima ($\Delta\lambda_{Em\text{-}max}$) of curves ±12-OPDA. Asterisks indicate significant difference ($p \leq 0.05$) in

comparison to control (0 µM 12-OPDA), calculated using one-way ANOVA with post hoc Tukey HSD. Right bottom graphic, plot of fluorescence intensities at λ_{Em-max} against 12-OPDA concentration. Results are means± SD of n≥3. Asterisks indicate significant difference ($p \leq 0.05$) in comparison to control, calculated using One-Way ANOVA with post hoc Tukey HSD.

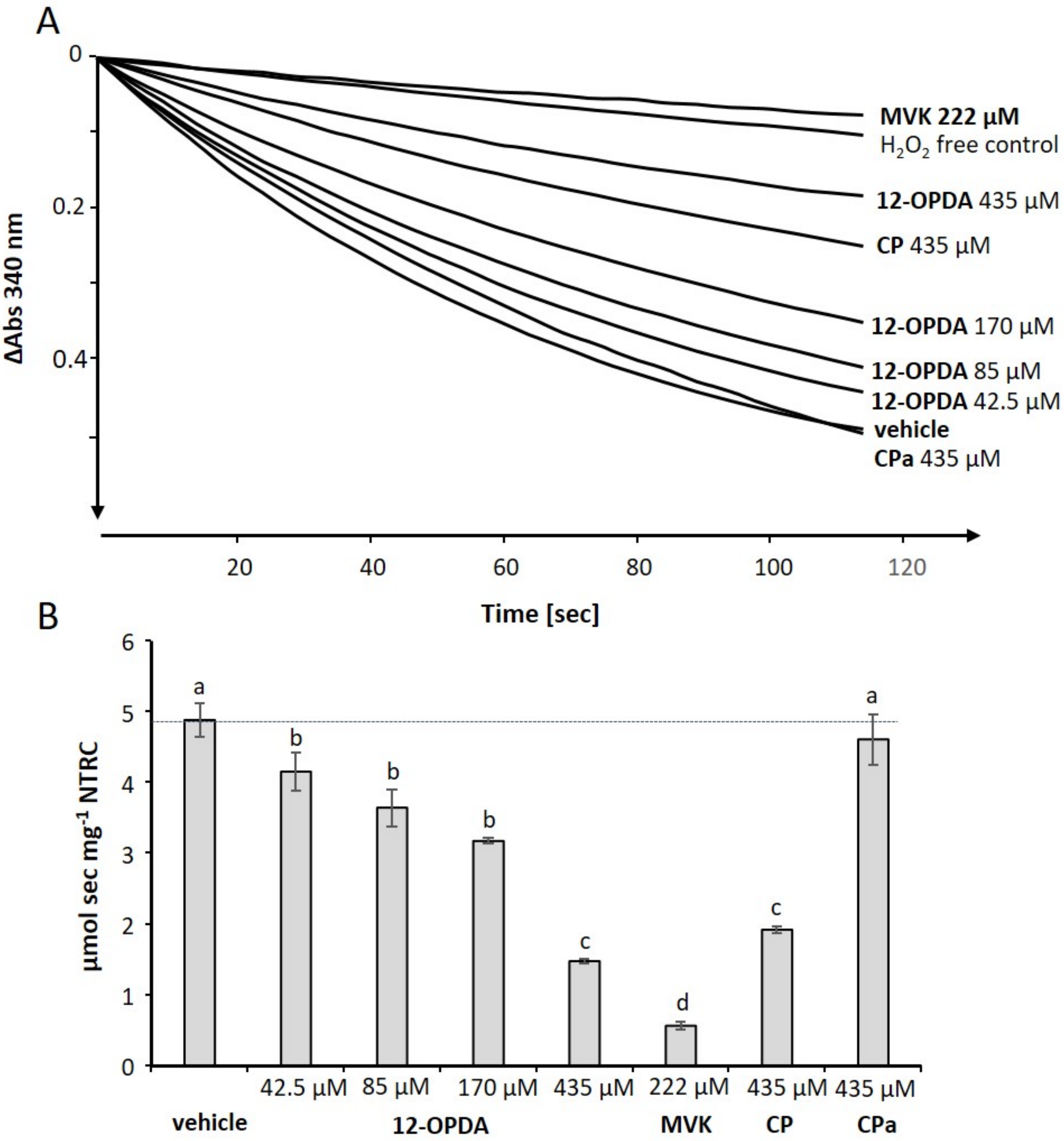

Figure S7. NTRC-coupled peroxidase activity of 2-CysPRX in presence of CCCs. (**A**) 121 µL 50 mM K-P_i, pH 7.5, containing the indicated molecules or solvent (EtOH), 30 µg NTRC, 10 µg 2-CysPRX, and 115 µM NADPH were incubated for 200 s followed by addition of 10 µL 980 µM H_2O_2 (75 µM) or the same volume of H_2O (H_2O_2-free control). Each curve is representative for 3-4 measurements. Additional controls were as follows, BSA instead of 2-CysPRX and buffer instead of NTRC showed only slight oxidation of NADPH similar to the curves MVK 222 µM and H_2O_2-free control. (**B**) Bar diagram of reactivation of 2-CysPRX by NTRC in presence of CCCs. Initial rates (first 20 s after addition of H_2O_2) of NADPH consumption by NTRC due to reactivation of 2-CysPRX, as shown in (**A**), were taken as 2-CysPRX activity. At the tested concentration of 42.5 µM 12-OPDA, the protein thiol-mediated reduction of H_2O_2 by 2-CysPRX was impaired. The effect was maximal in 435 µM 12-OPDA. Although not significant (p=0.2) compared to the same concentration of CP, 12-OPDA seemed to be more inhibitory, thus indicating specific involvement of

the cyclopentenone side chains of 12-OPDA in interference with the NTRC-2-CysPRX protein-interaction. MVK at a concentration of 222 µM strongly inactivated the activity, while 435 µM CPa had no effect on the activity of 2-CysPRX in the presence of NTRC. Values are means (n=3-4) ±SD. Significant differences were calculated using One-Way ANOVA with post hoc Tukey HSD and are represented at $p \leq 0.05$ by different letters.

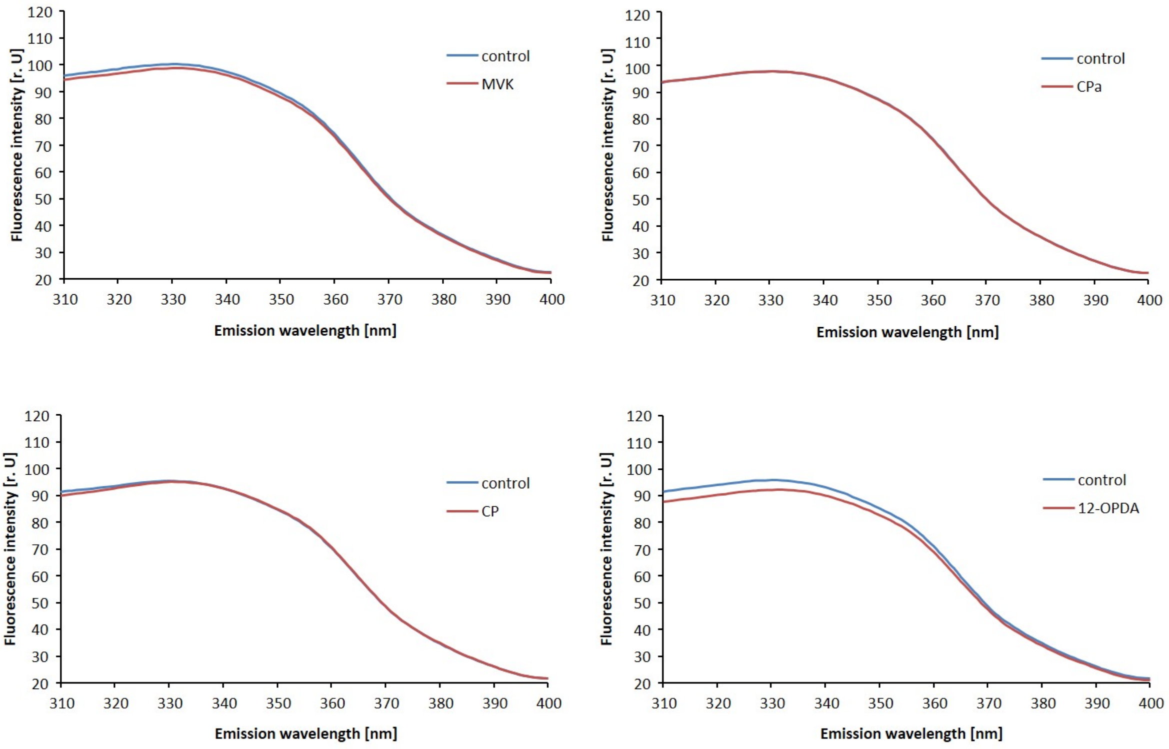

Figure S8. Effect of 12-OPDA and related compounds on the intrinsic fluorescence emission of Cyp 20-3**.** Intrinsic fluorescence analysis of Cyp 20-3 (30 µM) incubated with indicated compounds (125 µM) or solvent (control) was performed at least six times with identical results. Values see Table 3. Details see Materials and Methods.

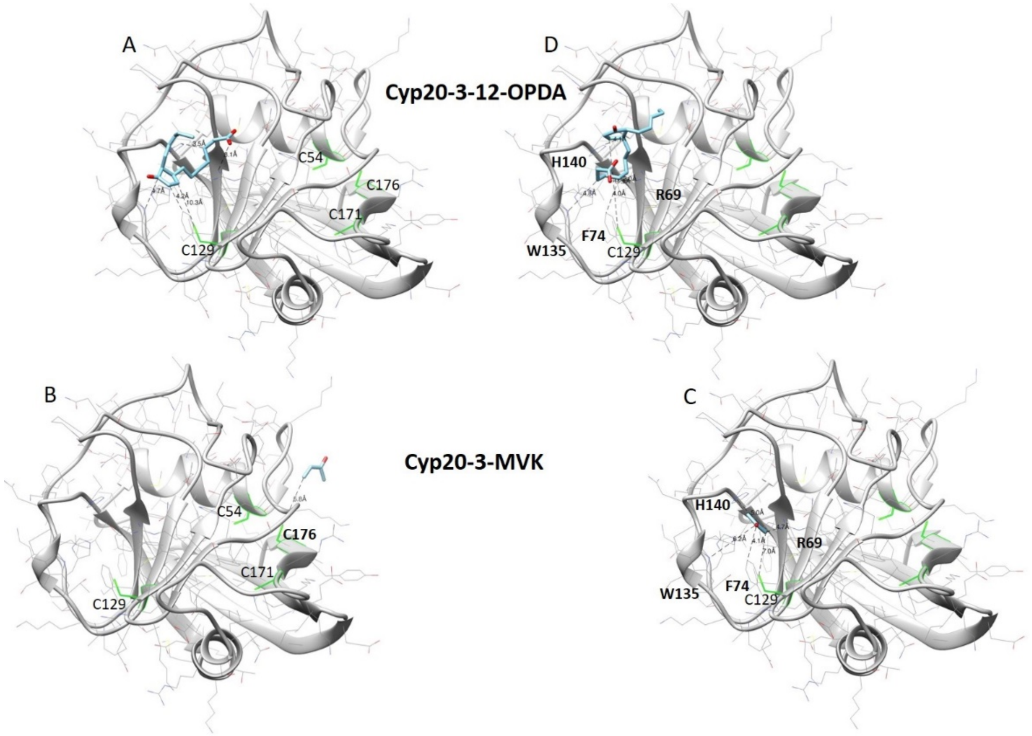

Figure S9. Docking study of Cyp20-3 with 12-OPDA and MVK. The tertiary structure of homology-modelled Cyp20-3 (residues 93-256), based on human Cyp PDB 5KUO, consists of eight β-strands and three α-helices. This computational docking experiment aimed to explore whether any of the four Cys residues of Cyp20-3 (C54, C129, C171, C176 in green) might be targeted by CCC (shown for MVK and 12-OPDA depicted as stick model) and to reveal the most probable ligand-binding grooves. Of all theoretical binding models obtained Cyp20-3s, sidechain C129 scored best in terms of closeness to the β -carbon of 12-OPDA (10.3 Å). (**A**) Depiction of interatomic distances (dashed line) towards C129, R69, W135, F74, and H140. Same as for C129, no other Cys residue was in the distance range below 6Å that could indicate binding of 12-OPDA to Cyp20-3 thiols (models not shown). This was not the case with MVK, as depicted in (**B**) MVKs terminal sp2-C might target the thiol of Cys 176 (interatomic distance 5.8 Å). Binding energy: -4.8 kcal mol-1. (**C**) Docking result of highest computed binding affinity of MVK to Cyp20-3 (-5.3 kcal mol^{-1}) suggests that C129 in comparison to Cys176 is less likely to be targeted by MVK as revealed by the interatomic distance of C129 S to the terminal ´ene´ carbon of MVK (7.0 Å). The contacts to the RWFH motif are highlighted. (**D**) In the solvent-accessible cavity spaced in between the smallest α-helix, the β-sheet bridging top loop, and the β-sheet, a 12-OPDA binding cleft is proposed that is constituted of side chain residues (RWFH highlighted) within a distance of 5 Å from 12-OPDA, interatomic distance of 12-OPDAs β-carbon to C129s thiol (11.8 Å) is also shown. Of all simulations (part results see Table S3) the presented binding mode (**D**) scored best, with a calculated binding energy of -8.5 kcal mol^{-1}. C and O atoms of ligands are shown in blue and red, protein backbone in grey ribbon, and side chain residues in stick representation (C in grey, O in red, and N in blue). Docking and visualization as in Figure 6A upper graphic.

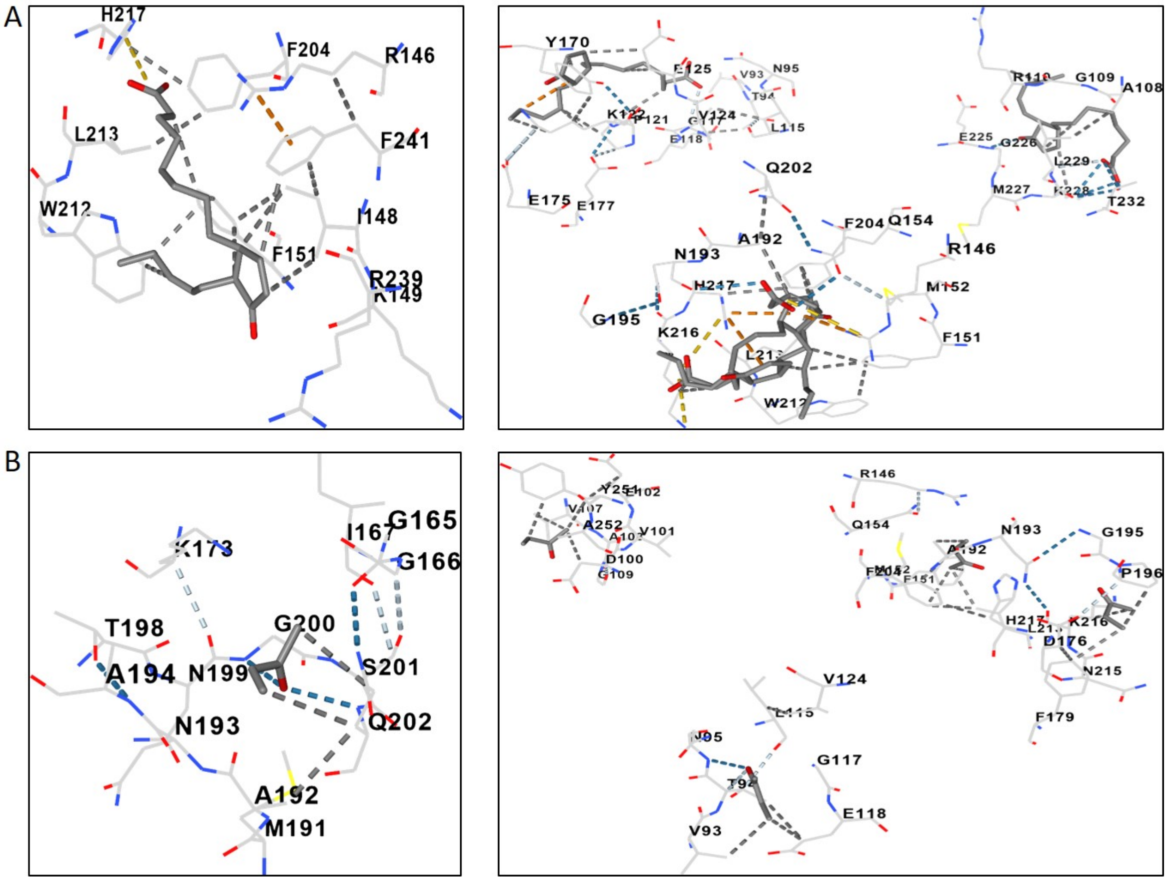

Figure S10. *In silico* interaction study of Cyp20-3 with 12-OPDA and MVK performed with CB-Dock. (**A**) Suggested 12-OPDA (shown in grey stick representation) binding cavities (sizes of 99 (left) and 141, 62, 56 and 83 right) with involved sidechains are shown as stick model. All five binding cavities are presented, with Vina scores of -5.1 (left) and -4.9, -4.7, -4.3 and -3.8 (right). Per default the online results of CB-Dock are presented with numbering referring to the full primary sequence and according to numbering from N-C-termini of Cyp20-3, RWFH by convention (Laxa et al., 2007 [32]) is 146, 212, 151 and 217. In this context Cys residues 54, 129, 171, 176 are numbered as 131, 206, 248, 253. (**B**) Suggested MVK (shown in grey stick

presentation) binding cavities (sizes of 144 (left) and 99, 62, 56 and 83 right) with involved sidechains are shown as stick model. All five binding cavities are presented, with Vina scores of -3.3 (left) and -3.3, -2.7, -2.7 and -2.5 (right). Residue numbering and depiction same as in (**A)**. To ease presentation, secondary structures are not depicted. Details see Materials and Methods.

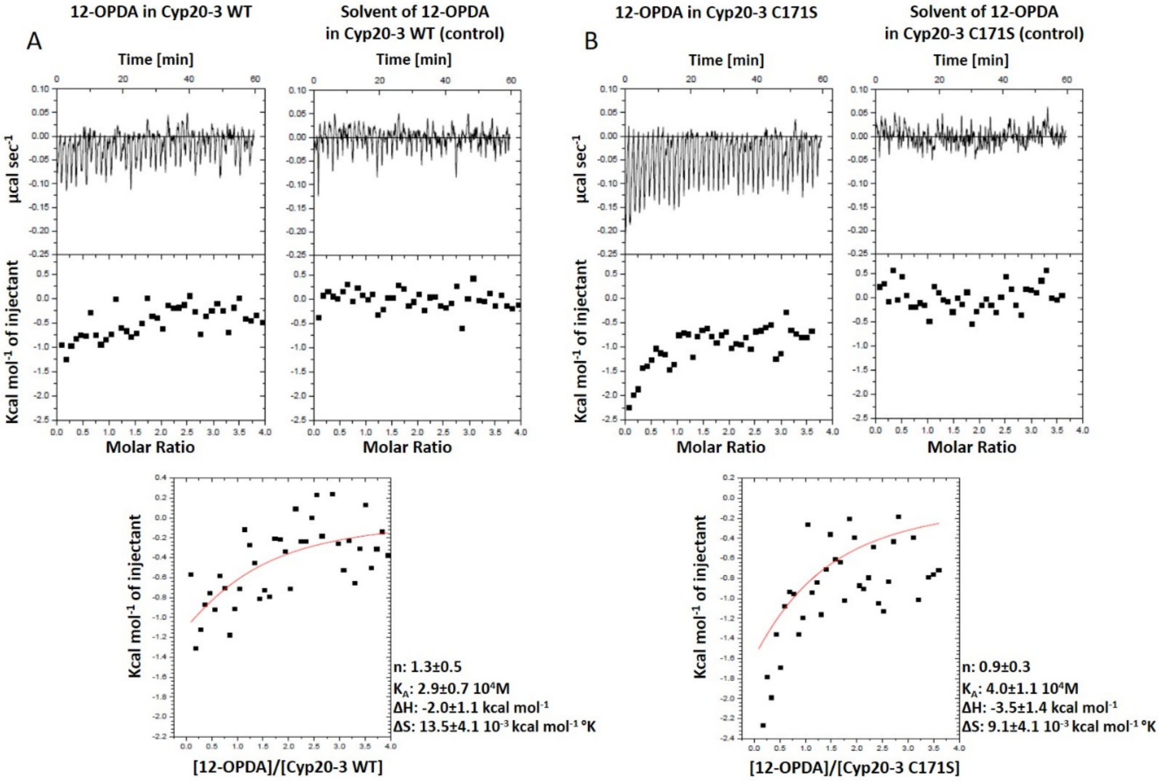

Figure S11. Calorimetric analysis of the 12-OPDA interaction with Cyp20-3 and Cyp20-3 C171S. Titrations (**A-B**) are representative of two determinations each. The final results (n=2±SD) with subtracted background as determined by injection of solvent (0.1 % EtOH in dialysis buffer) into dialyzed proteins are shown at the bottom. The red line repesents a binding site fit with Chi^2DOF of 8.3 10^4-1.2·10^5 to the obtained binding curve. Abbreviations: n: molar ratio; K_A: association constant; ΔH: reaction enthalpy; ΔS: change in entropy.
